# Evolution repeats itself in replicate long-term studies in the wild

**DOI:** 10.1126/sciadv.adl3149

**Published:** 2024-05-24

**Authors:** Patrik Nosil, Clarissa F. de Carvalho, Romain Villoutreix, Laura S. Zamorano, Marion Sinclair-Waters, Nicholas P. Planidin, Thomas L. Parchman, Jeffrey Feder, Zach Gompert

**Affiliations:** ^1^ Theoretical and Experimental Ecology (SETE), CNRS, 2 route du CNRS, 09200 Moulis, France.; ^2^ CEFE, Université de Montpellier, CNRS, EPHE, IRD, Montpellier, France.; ^3^ Departamento de Ecologia e Biologia Evolutiva, UNIFESP, Diadema 09972-270, Brazil.; ^4^ Department of Biology, University of Nevada, Reno, NV 89557, USA.; ^5^ Department of Biology, Notre Dame University, South Bend, IN 11111, USA.; ^6^ Department of Biology, Utah State University, Logan, UT 84322, USA.

## Abstract

The extent to which evolution is repeatable remains debated. Here, we study changes over time in the frequency of cryptic color-pattern morphs in 10 replicate long-term field studies of a stick insect, each spanning at least a decade (across 30 years of total data). We find predictable “up-and-down” fluctuations in stripe frequency in all populations, representing repeatable evolutionary dynamics based on standing genetic variation. A field experiment demonstrates that these fluctuations involve negative frequency-dependent natural selection (NFDS). These fluctuations rely on demographic and selective variability that pushes populations away from equilibrium, such that they can reliably move back toward it via NFDS. Last, we show that the origin of new cryptic forms is associated with multiple structural genomic variants such that which mutations arise affects evolution at larger temporal scales. Thus, evolution from existing variation is predictable and repeatable, but mutation adds complexity even for traits evolving deterministically under natural selection.

## Teaser

Natural selection and mutation shape how stick-insect evolution replays itself.

## INTRODUCTION

The extent to which evolution is repeatable and predictable is central to understanding the role of determinism and chance in the history of life, with implications for both basic and applied science (*1*–*4*). These ideas are captured in Gould’s famous metaphor of “replaying the tape of life” (*5*). Gould argued that historical contingency and chance idiosyncrasies would result in different (i.e., non-repeatable) evolutionary outcomes if the history of life was to be replayed over and over again. However, others such as Morris (*6*) have argued that evolution is inherently predictable, and many examples of deterministic natural selection promoting repeatable and predictable elements to evolution do exist (*3*, *7*). Thus, beyond spurring decades of debate, Gould’s metaphor has been usefully transformed into an empirical research program (*1*, *8*).

Comparative phylogenetic studies of parallel evolution often support repeated outcomes driven by natural selection (*1*, *7*, *9*). However, inferring evolutionary processes and their interplay from such retrospective work can be challenging. For example, selection that fluctuates rapidly between time points can be misconstrued as evolutionary stasis if only end outcomes, or a few distant time points, are analyzed. Detailed studies of the fossil record and ancient DNA can help address these issues (*10*, *11*), as can real-time studies of evolutionary dynamics. The latter are exemplified by studies of experimental evolution in replicated laboratory populations of microbes (*12*–*14*) and other organisms, such as insects (*15*, *16*). This impressive body of work has revealed not only repeatable patterns of evolution by natural selection but also a role for the contingency of mutation.

**Fig. 1.**
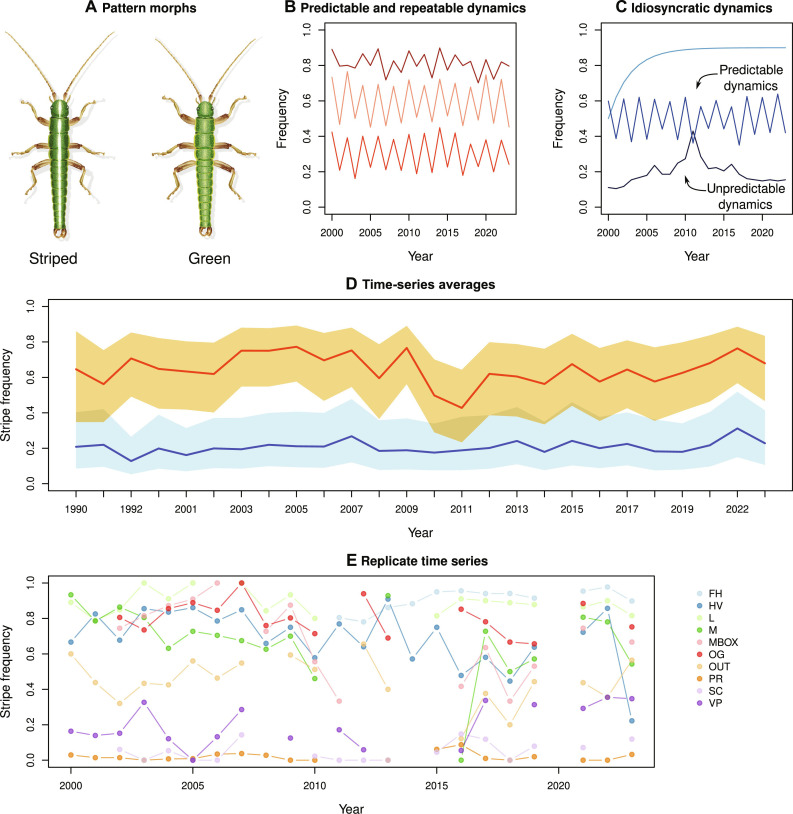
Evolution in replicate long-term field populations of *T. cristinae* stick insects. (**A**) Illustrations of the color-pattern morphs of *T. cristinae*. (**B**) Predictions of highly repeatable evolutionary dynamics over time. Each line represents a different population, each exhibiting predictable “up-then-down” fluctuations in trait or gene frequency over time. (**C**) Predictions of less repeatable evolutionary dynamics over time. In contrast to the panel to the left, each population exhibits different patterns of trait or gene frequency change over time. Note the distinction between predictability within any single time series (i.e., population or replicate) and repeatability among them. (**D**) Empirical variation in morph frequencies in *T. cristinae* between 1990 and 2023. The orange line (median) and shading [95% equal-tailed probability interval (ETPI)] represent yearly averages on the host plant *Adenostoma*. The blue line (median) and shading (95% ETPI) represent yearly averages on the host plant *Ceanothus*. (**E**) Population-specific morph frequency variation over time, representing replicate evolutionary dynamics (mean number of years per population = 14). Results are shown for the 10 core populations, i.e., replicates, that this study focuses on.

In contrast to laboratory experimental evolution studies, replicated, long-term studies from natural populations are rare. Certainly, highly influential long-term studies of the predictability of evolution in the wild do exist, in finches and other birds (*17*–*20*), moths and butterflies (*21*–*23*), stickleback and guppy fish (*24*–*27*), sheep (*28*), fruit flies (*29*), and deer (*30*) (to name a few). For example, rare climatic events affect evolution in the famous Darwin’s finches (*19*, *20*). However, most such work is restricted to one or few populations, making it difficult to draw inferences on repeatability among multiple evolutionarily independent populations (note the distinction between predictability within a population and repeatability among populations, Fig. 1). Thus, replicated studies of evolution in the wild are required to test the generality of the findings from microbes (*12*–*14*) and to help bridge now disparate laboratory and field studies. Such studies are challenging to implement not only because they take concerted effort but also because time cannot simply be sped up with more effort.

**Fig. 2.**
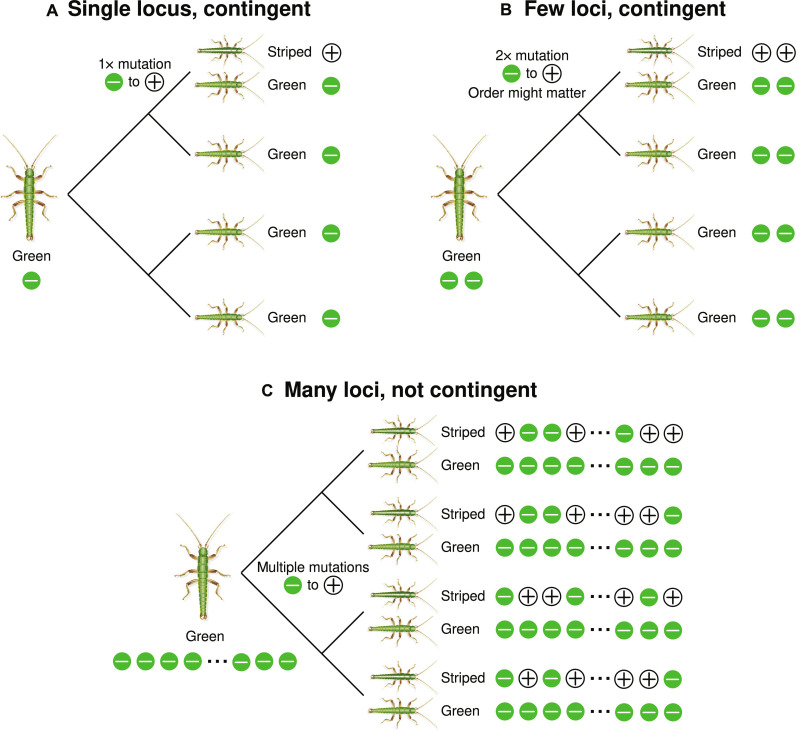
Conceptual diagram illustrating the relationship between the genetic architecture of a trait and the repeatability of evolution at the genetic and phenotypic levels (where the tips of each phylogeny represent a different taxon/species). (**A**) Hypothetical scenario where a trait (i.e., stripe) depends on a specific mutation. In this case, the evolution of stripe is contingent on the specific mutation occurring and thus occurs only once in the hypothetical evolutionary scenario depicted on the phylogeny. Thus, phenotypic evolution is not repeatable (it would, however, have a repeatable genetic basis if the trait evolved in multiple species). (**B**) An alternative scenario where stripe depends on a few loci. Here, the evolution of stripe is still contingent on a small number of specific mutations occurring and this might make the trait evolve less repeatedly. Moreover, in such cases, evolution might (or might not) be dependent on the order in which the mutations occur (i.e., some mutations might only be beneficial if another mutation has already occurred). (**C**) A third alternative scenario where stripe is affected by many mutations (i.e., is polygenic). In this case, stripe does not depend on a specific, unlikely mutation, and is unlikely to depend on the order of mutations. This genetic architecture thus makes the repeated evolution of stripe much more likely, occurring in all four species in the hypothetical example. However, the genetic basis of stripe varies among the species, and, thus, the genetic basis of stripe is not repeatable (different combinations of alleles, i.e., mutations, can generate a stripe). In all diagrams, + and − symbols denote alternative alleles, with + contributing to the stripe phenotype.

Here, we provide such a study on the basis of compiled data from 30 years of tracking morph frequencies across 10 replicate populations of a stick insect in the wild (Fig. 1). We integrate these data with field experiments, modeling, and genomic data to elucidate the processes driving evolutionary dynamics across timescales. In particular, our genomic analyses allow consideration of the contingency of mutation, which can add complexity and nuance relative to the sole consideration of evolution from standing genetic variation (*31*). Specifically, mutational dynamics can be related to the genetic architecture of traits, with consequences for the repeatability of evolution at the genetic versus phenotypic level (*32*, *33*). Some predictions are as follows and illustrated in Fig. 2.

When traits are controlled by simple genetic architectures composed of one or few loci of large effect, then individual mutations can strongly affect evolutionary dynamics (*4*, *34*). In such cases, individual mutations “matter” and mutation can be an important consideration for understanding evolution. For example, such architectures offer few mutational targets, making mutations that improve fitness rare (*4*, *34*). Moreover, if there are strong interactions between loci (i.e., epistasis), then only a few genetic combinations may work well together to increase fitness. In other words, there are few mutational sequences or “paths” that adaptive evolution can actually take (*4*, *34*–*36*). Thus, traits controlled by few loci may be challenging to evolve de novo and thus show modest repeatability in their origin over time. However, when such traits do evolve, they will have a predictable, repeated genetic basis (i.e., using the same, few loci and mutations that increase fitness). These predictions could be different for more polygenic architectures composed of many loci with smaller effects (*37*). Here, no individual mutation has a strong effect on a trait, many loci have redundant effects, and path dependency is unlikely to force a particular mutational sequence to be used to evolve higher fitness. Thus, traits controlled by many loci may evolve repeatedly because they can use a wide range of mutational variation. However, when they do so, a different set of loci and mutations is likely to be used in each instance, making evolution at the genetic level not very repeatable (*37*).

We acknowledge that there can be much nuance beyond the stylized ideas and predictions noted above [e.g., see (*38*)]. However, these ideas, nonetheless, illustrate why it can be informative to study the genetic architecture of traits that are being analyzed for their repeatability, particularly to understand the dynamics of evolution from standing variation versus new mutation. In this context, we here combine our time-series and experimental work with genomic analyses of the genetic architecture of cryptic color pattern. Our results reveal clear similarities but also differences with past work focused on body color (rather than pattern, see below for details). Our integrative approach leads to a more complete understanding of the repeatability of evolution than would be possible by studying selection or mutation in isolation.

### Ecology and genetics of *Timema* stick insects

Our study system is *Timema* stick insects (Fig. 1), a genus of wingless, plant-feeding insects found throughout southwestern North America (*39*). We focus primarily (but not exclusively) on *Timema cristinae*, which exhibits three highly heritable morphs and uses two primary host plant species (*40*, *41*). Two of these morphs have diverged in frequency between host species due to strong divergent selection imposed by visual predators such as lizards and birds (*42*, *43*). Specifically, a green unstriped color-pattern morph (green morph hereafter) is cryptic on the broad leaves of *Ceanothus*. In contrast, the striped color-pattern morph (a green morph that also bears a white, longitudinal stripe on its dorsal surface) is cryptic on the thin needle-like leaves of *Adenostoma*. Thus, each morph is generally more common on the host on which it is more cryptic, which led to a body of past work on the potential for divergent adaptation between hosts to drive ecological speciation (*41*, *44*).

However, we stress that polymorphism is maintained such that both green and striped morphs occur in most populations. This variation is maintained due to negative frequency-dependent selection (NFDS) and gene flow, creating a mosaic of variation in morph frequencies across the landscape, both within and among populations (*45*, *46*). Specifically, the frequency of the striped morph varies within local populations ranging from low (near zero), to intermediate, to high (near one) but is rarely truly fixed (Fig. 1). This provides the requisite variation to study changes in the frequency of green versus striped morphs over time, which we focus on here. There is also a third darkly colored or melanic morph that is rarer and found at comparable frequencies between the two hosts. The melanic morph is not specifically adapted to either host (*40*, *47*) does not fluctuate strongly in frequency over time and does not appear subject to NFDS (*40*, *45*, *47*). Thus, we focus first on testing for repeatable fluctuations in color-pattern morphs but return to melanism later in this study when considering longer timescales. We also return to the relationship between polymorphism and speciation at the end of our study.

Past observations and a manipulative field experiment documented changes in color-pattern morph frequencies that were highly predictable due to NFDS (i.e., a fitness advantage to rare forms) (*45*). This likely occurs due to birds switching search images to hunt for common prey items (*48*–*51*). Thus, increases in the frequency of the striped morph one year were reliably followed by decreases the following year and vice versa. This past work focused on changes in a single 18-year time series (i.e., a single population or “play of the tape”) because substantial long-term data from other populations did not exist at the time. Thus, past work could not address the issue of repeatability or “replays,” i.e., among replicate populations, per se. We thus focus here on the repeatability of evolution among populations.

We report results from 30 years of data collection on morph frequencies in *T. cristinae*, representing 692 year-by-host-by-locality estimates of morph frequency derived from 48,349 individuals (Fig. 1 and data S1). Most critically, these data represent 10 localities with at least a decade of the data required to test for NFDS (see table S1; mean, 14 years; maximum, 22 years). These 10 localities were chosen to represent replicates, for example, because of little to no gene flow among them such that each locality undergoes independent yearly changes in morph frequency. Supporting this claim, we emphasize that our 10 localities are geographically separated (fig. S1), generally by several kilometers, yet the average per-generation dispersal distance based on a mark-recapture study is only 12 m (*52*). This makes it very unlikely that there is sufficient migration between our localities to generate detectable morph frequency fluctuations. Molecular data further support limited gene flow between our localities and thus their evolutionary independence (especially in the context of yearly changes in morph frequency). For example, genetic data have shown that even parapatric populations that are directly adjacent to one another exchange only a few migrants per generation, and all of our study localities here are geographically separated from each other such that gene flow among them is even lower (fig. S1) (*53*, *54*). Thus, each locality acts as a replicate or replay for analyzing the repeatability of evolution, particularly because the distance (i.e., kilometers) typically separating each locality make it likely that different bird and lizard individuals hunt insects at each locality.

Notably, *T. cristinae* is univoltine with nonoverlapping generations. Thus, each year of data represents a generation, with evolution occurring between each pair of years (the insects diapause as eggs through autumn and winter, hatch in spring, and mate and die in early summer, repeating this cycle each generation). Despite these data representing an appreciable effort, the timescales involved are very different from those associated with Gould’s metaphor. We thus also consider mutations affecting color pattern over deeper evolutionary time (*55*).

Here, the genetic architecture of color pattern is relevant, for reasons outlined above and in Fig. 2. Specifically, we here evaluate the extent to which stripe depends on specific mutations that could make the repeated evolution of the trait less likely. Past work suggests that pattern is controlled by one or few loci on chromosome 8 (chr8 hereafter) (*40*, *47*, *56*). However, the number, effect sizes, and physical distributions of the loci involved remains unclear. Specifically, past work described a region of chr8 named the *Mel-Stripe* locus that harbors complex structural variation that explains most of the variation in body color (a large inversion and deletion distinguish green versus melanic morphs, indicative of suppressed recombination and opening the potential for linked selection) and is also partially associated with color pattern (striped versus green). However, whether one or multiple regions of *Mel-Stripe* or even other loci are associated with pattern remains unclear due to (i) the focus of past work on color (not pattern) and (ii) the use of a fragmented reference genome. Here, we integrate better chromosome-level genome assemblies with genome-wide association (GWA) mapping to show that color pattern is actually associated with multiple different structural variants within the *Mel-Stripe* locus, as well as a chromosomal inversion in a region not associated with color or pattern in past work (i.e., the “*Pattern*” locus, which is reported here for the first time). We conclude our study by discussing what these genetic details tell us about the repeatability of evolution.

## RESULTS

### Highly repeatable fluctuations among replicate populations

**Fig. 3.**
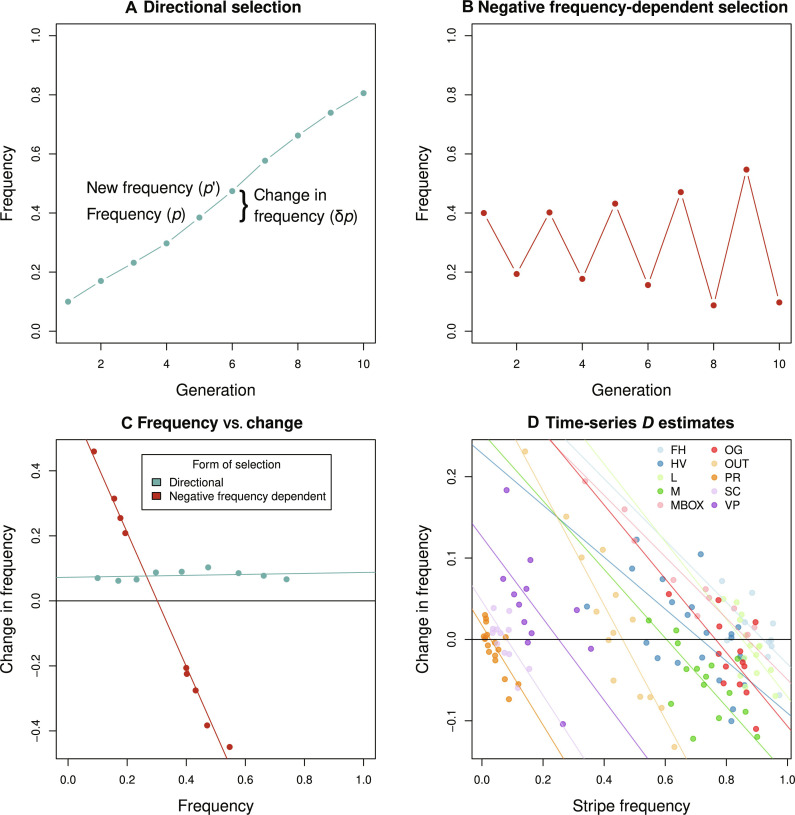
Estimates of the NFDS parameter *D* and its repeatability among the study populations. (**A**) Hypothetical time series under directional selection, also illustrating current and subsequent morph frequencies, and how the difference between them is used to estimate the parameter *D*. (**B**) Hypothetical time series under NFDS. (**C**) Predictions for the relationship between *p* and Δ*p*, under directional selection versus NFDS (blue versus red, respectively). A strongly negative association is expected under NFDS when *p* is close to $p=\hat{p}$. For more quantitative theoretical expectations, see Fig. 5. (**D**) Replicate time-series data in *T. cristinae* reveal evidence for NFDS. Specifically, shown is stripe morph frequency versus change in stripe frequency between successive time points, for each study locality. Under NFDS, a negative association is predicted, as observed in all 10 populations studied here. The value where the fit line crosses the zero point on the *y* axis (denoted by a horizontal line) is the predicted equilibrium frequency under NFDS (see fig. S1). For raw data, see Fig. 1.

Analyses of 30 years of field data revealed fluctuating frequencies of the striped versus green morphs over time in all 10 replicate populations (Fig. 1). Thus, decreases in the frequency of the striped morph in one year tended to be followed by increases the following year. A past field experiment and time-series data from a single locality demonstrated the existence of NFDS in our study system (*45*). We thus assume that NFDS contributes to these fluctuations but in conjunction with other processes as analyzed in detail below. In this context, NFDS can be quantified by the parameter *D* (*57*), defined as the linear correlation between *p* (in our case, the frequency of the striped morph) and the change in *p* from the previous time point (Δ*p*) when the system is evaluated near the equilibrium, $p=\hat{p}$ (i.e., where the relationship between *p* and Δ*p* is approximately linear). A negative *D* is predicted under NFDS (Fig. 3), as introduced by Lewontin in the 1950s and used by others (*57*–*59*).

We found that *D* was negative in all 10 replicate populations, consistent with repeatability among populations (Fig. 3 and table S1). Although *D* did vary (SD, 0.12 among replicates), it was consistently negative without extreme variability (mean *D*, −0.48; range, −0.70 to −0.32). Thus, fluctuations indicative of NFDS, replicated in each population, are a repeatable feature of the system. Notably, we detected negative *D* even in populations exhibiting limited variation, hinting at powerful sources of NFDS and strong repeatability. Our data further allowed us to quantify the equilibrium frequency around which evolution by NFDS fluctuates within each locality. In this context, we found a strong correlation between the predicted equilibrium frequency and the observed mean frequency (Pearson correlation, *r* = 0.99, *P* < 0.001) (fig. S2). Equilibrium frequencies varied substantially among the 10 replicate populations (fig. S2). Consistent with past work (*46*, *60*), nearly all of this variability in predicted equilibrium frequencies among the 10 *T. cristinae* localities was explained by variation in the relative abundances of the two hosts plants, *Adenostoma* and *Ceanothus*, at each locality (linear regression, model *r*^2^ = 0.93, *P* < 0.001) (fig. S3).

### The observed fluctuations are not converging to an equilibrium

**Fig. 4.**
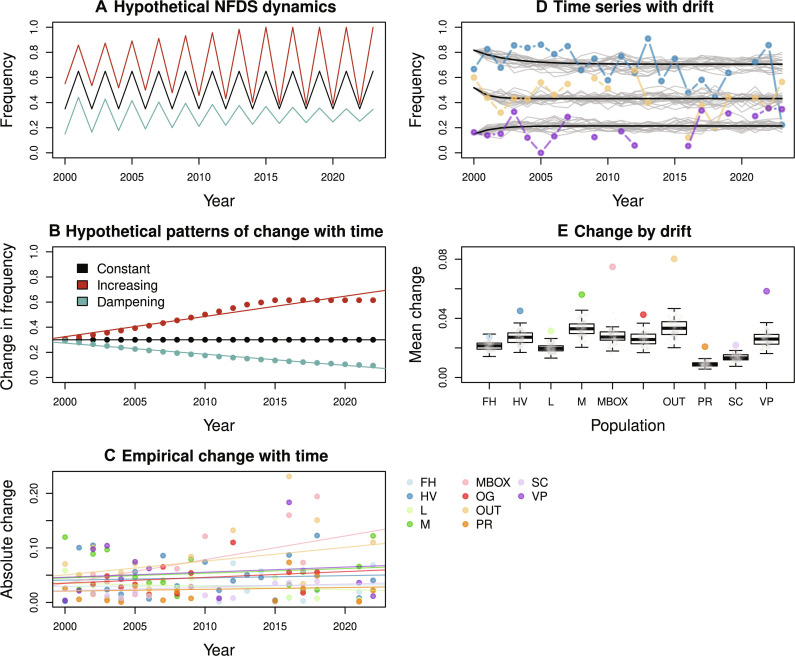
Alternative evolutionary dynamics with NFDS, and simulations quantifying the extent to which perpetual fluctuations with NFDS can arise due to external forcing by genetic drift. (**A**) Possible NFDS dynamics with constant, increasing, or dampening oscillations over time. These lead to different relationships of the absolute change in frequency (e.g., stripe frequency) as a function of time; points and a best-fit line are shown for these hypothetical examples in (**B**). (**C**) A scatterplot showing the absolute change in stripe frequency for the empirical stripe frequency data, for the 10 localities as a function of year. Colored lines are best-fit lines for each location from a hierarchical Bayesian linear regression, colored to denote localities as in Fig. 1. The analysis provides no evidence of a decline in the magnitude of stripe frequency change over time in any location (if there is any credible change, then it tends to be positive; see main text). (**D**) Three representative empirical time series (colored dots and lines) and year-to-year fluctuations expected theoretically under NFDS and drift (i.e., no other external forcing, gray lines). (**E**) Median observed fluctuations for all 10 replicate populations (colored dots) and expected change under NFDS plus drift (box plots show the median and first and third quartiles of the null distribution with whiskers extending to the minimum and maximum or 1.5 times the interquartile range; points denoting each null simulation are overlain on the box plots). The examples in (D) and full results in (E) depict how the observed magnitude of fluctuation is generally greater than expected under NFDS plus drift. This implies external forcing via gene flow or an additional source of selection on color pattern, perhaps related to the association of color pattern with structural genomic variation such as inversions.

Although our time-series results are consistent with NFDS, other phenomena, such as genetic drift, gene flow, and forms of selection other than NFDS, can contribute to fluctuating patterns such as those reported here (*57*, *58*, *61*, *62*). Past theoretical work has shown that NFDS alone does not always generate perpetual fluctuations (*58*). Specifically, the *D* values observed in our time-series results (e.g., −0.70 to −0.32) are in the range expected from theory for NFDS alone to lead to dampened oscillations and convergence to an equilibrium, rather than long-term fluctuations (*58*). However, we found no evidence in our empirical time series for such convergence or for a decline in the magnitude of frequency change over time [hierarchical Bayesian model, posterior probability (pp) the mean effect of time is less than 0 = 0.11; two populations have evidence of increases in change over time with pp = 0.98, whereas none exhibited credible evidence of decline, all with pp less than 0.71; see table S2 and Fig. 4. We thus next focused on the causes of the repeatable patterns of *D* (i.e., similar values of *D* and a lack of dampening oscillations), starting with theoretical and statistical considerations and then turning to additional data.

### Theoretical and statistical considerations help understand the causes of fluctuations

**Fig. 5.**
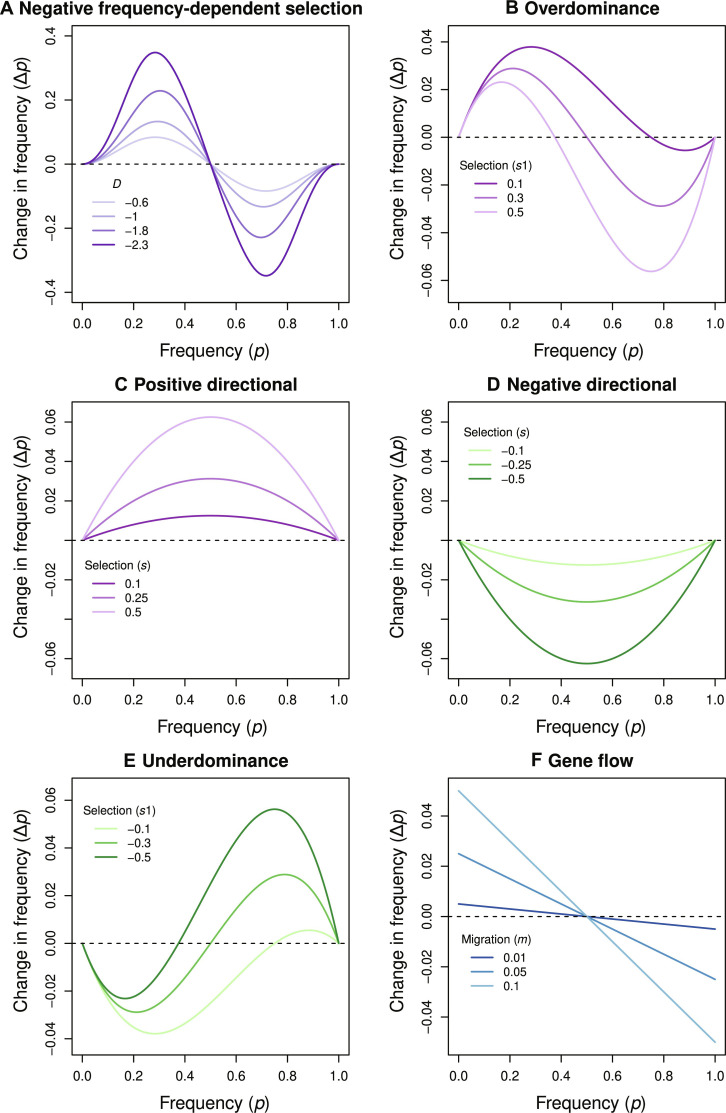
Theoretical expectations for evolutionary change under NFDS, other forms of selection, and gene flow. Panels show how evolutionary change (Δ*p*) depends on the allele frequency under various evolutionary processes. All results are based on standard analytical theory without genetic drift (*96*, *97*). Our focus here is on qualitative patterns of change (negative versus positive), not the magnitude of change per se, which depends on the intensity of selection. (**A**) NFDS with a range of values of *D* that would give rise to different evolutionary dynamics (see the “NFDS theory” section in the Supplementary Materials for analytical details). (**B**) Overdominance with Δ*p* = *p* * (1 − *p*) * [*s*2 − *p* * (*s*1 + *s*2)]; results are shown for *s*2 = 0.3 and a range of values of *s*1. (**C** and **D**) Directional selection such that Δ*p* = s * *p* * (1 − *p*) * [*p* + *h* * (1 − 2 * *p*)], with positive (C) or negative (D) values of *s* (the heterozygote effect, *h*, is fixed at 0.5). (**E**) Underdominance with Δ*p* = *p* * (1 − *p*) * [*s*2 − *p* * (*s*1 + *s*2)]; results are shown for *s*2 = −0.3 and a range of values of *s*1. (**F**) The effects of gene flow under a mainland-island model, such that Δ*p* = [*p* * (1 − *m*) + *p*_m_ * *m*] − *p*. Here, *p* and *p*_m_ are the island (focal population) and mainland (source) allele frequencies and *m* is the migration rate. These analytical results show that Δ*p* and *p* can exhibit positive, negative, or flat relationships for many evolutionary processes depending on the initial conditions and portion of an evolutionary trajectory analyzed. With NFDS, *p* is expected to be near the internal equilibrium where the relationship between *p* and Δ*p* should be negative.

Simple analytical theory illustrated in Fig. 5 demonstrates that, although some of the same data are used to estimate *p* and Δ*p* (i.e., Δ*p* = *p′* − *p*) (as well as the observed and predicted frequencies), a strong negative association (i.e., highly negative *D* value) between the two is not a given. For example, a strong negative association does not necessarily arise under positive directional selection (Fig. 3C). The theoretical results, coupled with past experimental evidence for NFDS (*45*), indicate that our time-series results concerning *D* are unlikely to represent a statistical artifact due to an inevitability of autocorrelation. The remaining challenge is thus to understand the combination of NFDS and other evolutionary processes that best explains our results.

**Fig. 6.**
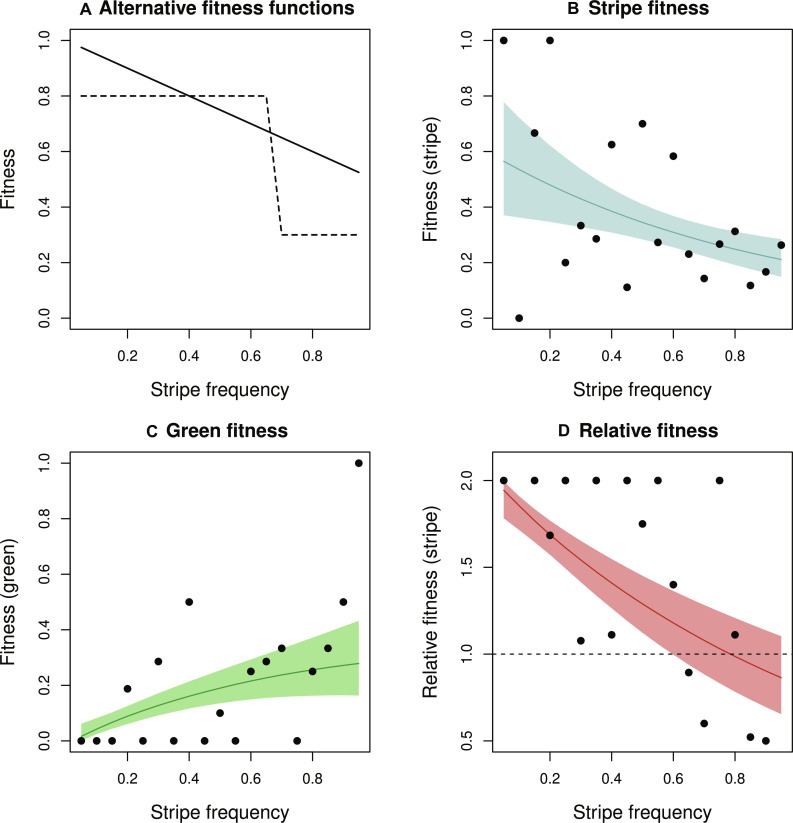
A field experiment demonstrates a linear NFDS fitness function. (**A**) Possible NFDS fitness functions, with perpetual fluctuations by NFDS alone being more readily maintained with the stepped function than a linear one. (**B**) Fitness of the striped morph as a function of striped morph frequency in an experimental transplant. (**C**) Fitness of the green (i.e., unstriped) morph as a function of striped morph frequency in the experimental transplant. (**D**) Relative fitness of striped versus green morphs as a function of stripe frequency. Fitness declines with increasing stripe frequency, as predicted under a model of NFDS. Note that the fitness function is relatively linear. In (B) to (D), dots represent values for each experimental bush with lines and shading representing the estimated fitness function [medians and 95% equal-tail probability intervals (ETPIs), respectively].

Here, the functional form of NFDS may be important to understand why fluctuations do not converge to an equilibrium. For example, if the NFDS fitness function is stepped and “threshold-like,” then selection suddenly reverses in direction, which might facilitate fluctuations through time in phenotype or genotype (Fig. 6) (*61*, *62*). We designed a field experiment to test this prediction, as reported below. Moreover, even with a linear fitness function (i.e., a linear relationship between morph frequency and fitness), theory predicts that fluctuations occur if NFDS is combined with other “external forces” such that there is continual overshooting of the equilibrium, followed by return toward it via NFDS (*58*). This forcing could stem from demographic variability and genetic drift, sources of selection other than NFDS, or a combination of the processes. Other possible sources of selection include environmental factors and associative overdominance (i.e., heterozygote advantage arising from different deleterious alleles accumulating on different structural variant genetic backgrounds) (*63*). Such additional sources of selection could generate linked selection (via linkage disequilibrium) on color pattern. We use simulations and genetic data to evaluate some of these possibilities, as reported below. We emphasize that these sources of forcing are not alternatives to NFDS; rather, they may be explanations for why ongoing fluctuations under NFDS are observed.

### A field experiment reveals a linear NFDS fitness function

We used a field transplant experiment to estimate the functional shape of the NFDS fitness function (e.g., stepped/sigmoidal versus linear). Past work in *T. cristinae* tested for NFDS using an experiment that transplanted green and striped morphs at two frequencies (20 and 80% striped) (*45*). By virtue of using only two frequencies, this past work could not test for a stepped versus linear fitness function. Here, we designed a field recapture experiment that increased the frequency of the striped morph among experimental *Adenostoma* bushes in 5% increments, ranging from 5 to 95%. In this manner, we estimated a fitness function on the basis of recapture rates, which are known to be a good proxy for survival in this system (*42*, *64*, *65*).

The results revealed that the relative fitness of the stripe morph declined gradually with increasing morph frequency (Fig. 6). This pattern provides unambiguous, direct evidence of NFDS and is indicative of an approximately linear NFDS fitness function, with no evidence for a stepped function (see also table S3 and fig. S4 for model comparison). Notably, the predicted equilibrium frequency of the striped morph based on the experiment is 75% (Supplementary Materials), and the observed mean frequency among all populations on *Adenostoma* is close to this value (70%) (Fig. 1). Thus, these experimental results once again support predictability in evolution by showing that morph frequencies in nature can be estimated from survival in a short-term experiment. However, given the values of our estimates of *D* from nature, and the fact that the fitness function from the experiment was smooth and approximately linear (i.e., not stepped), what prevents convergence to an equilibrium and the loss of marked fluctuations? In other words, what processes combine with NFDS to help maintain fluctuating dynamics across the 10 replicate populations?

### Selective and demographic variability are required to explain perpetual fluctuations

It is possible that random changes via genetic drift perturb populations away from the equilibrium strongly enough to facilitate perpetual fluctuations via NFDS (Fig. 4). We used a combination of genomic results and computer simulations to test this hypothesis. Specifically, allele frequency changes from genome-wide DNA sequencing at different time points can be used to estimate local effective population size (*N*_e_). Past work in *T. cristinae* using this approach estimated *N*_e_ to be 110 [95% equal-tailed probability interval (ETPI) = 106.8 to 113.7] (*66*, *67*). We conducted simulations of NFDS and genetic drift, conditioned on a *N*_e_ of 110. This revealed that fluctuations of the magnitude that we observed empirically are unlikely to arise by drift and NFDS alone (Fig. 4), implying that other demographic or selective processes are involved. For example, the mean change in stripe frequency per generation ranged from 1.3 to 2.7 times higher in each population relative to drift expectations (χ^2^ = 89.91, df = 20, *P* < 0.0001, Fisher’s combined probability test; see table S4). Moreover, mean change was greater in all populations than expected from the drift simulations (*P* for each population ≤ 0.03). A sensitivity analysis revealed that *N*_e_ would need to be below 40 before drift could begin to explain fluctuations of the magnitude we observed (fig. S5).

Thus, although drift may contribute to maintaining the observed fluctuations, it is unlikely to be the sole process interacting with NFDS to do so. Rather, unknown sources of selection, either acting on color pattern or on the genomic region affecting color pattern (e.g., linked selection), are likely involved, as well as contributions from local gene flow. Exogenous selective possibilities that warrant further study include climatic variation (*68*), variability in the predator community, and eco-evolutionary feedback loops that cause subtle but ongoing change (*69*).

The combined results to date support repeatable elements to evolution from standing genetic variation and provide insight into the processes involved. However, Gould’s metaphor is generally associated with longer timescales, where the contingency of mutation can affect evolution (*1*, *5*). To balance our study and explore this issue of timescale, we next turn our attention to the question of mutation. We do so by first elucidating the genetic architecture of color-pattern variation within *T. cristinae*, which sets the stage for then considering how mutations affect color pattern at timescales much deeper than those covered in our time series.

### Genetic architecture of color-pattern variation in *T. cristinae*

**Fig. 7.**
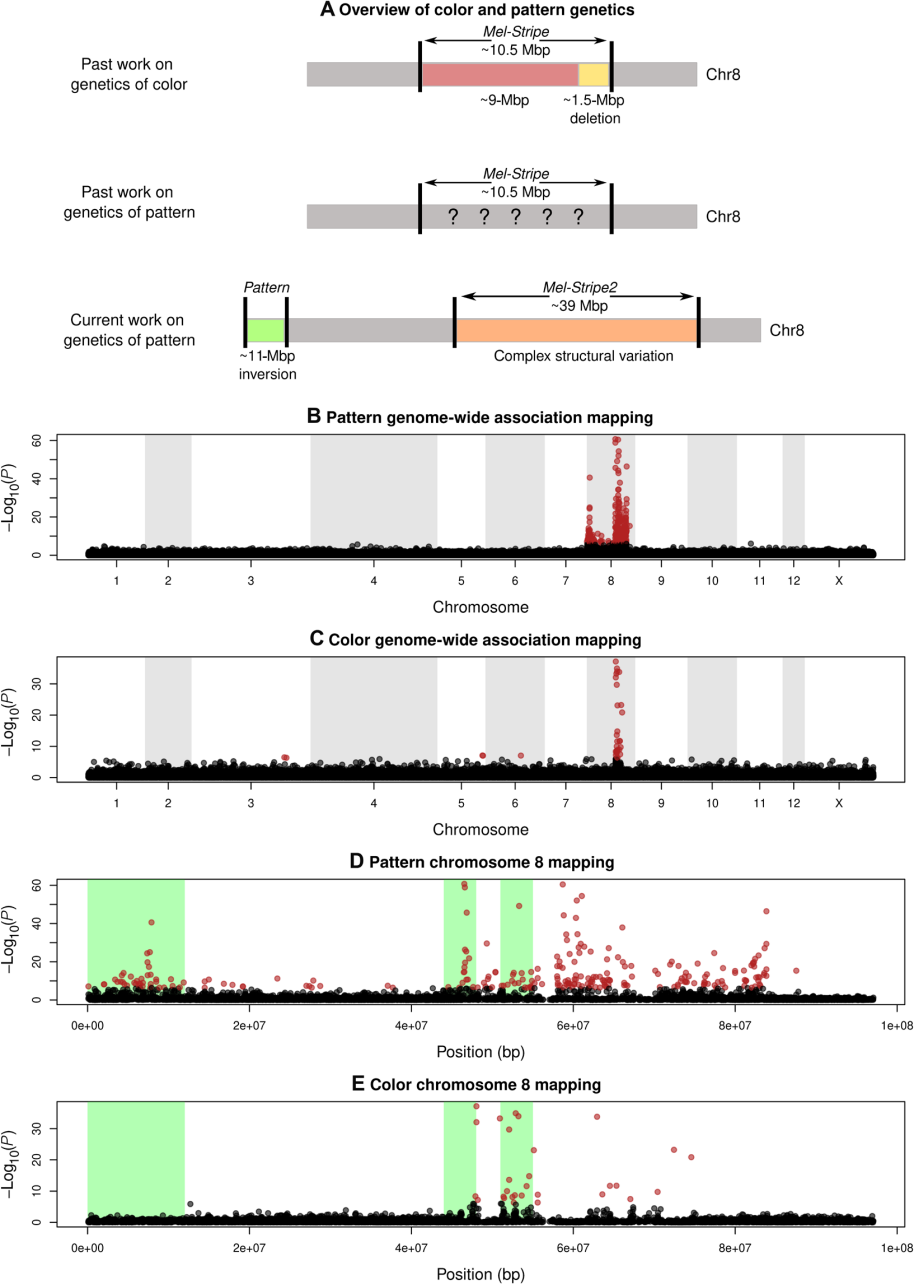
Genomic basis of the pattern (striped versus green) and color (green versus melanic). (**A**) Schematic cartoons highlighting past work on the genetics of pattern and color, relative to the discoveries concerning pattern in the current study. (**B**) GWA mapping of color pattern in *T. cristinae*. Shown is the −log_10_ *P* value from the null hypothesis test of no association along the 13 chromosomes in the genome. Note the two peaks of association on chr8. (**C**) GWA mapping of color (green versus melanic) along the 13 chromosomes in the genome. (**D**) GWA results for color pattern zoomed in on chr8. (**E**) GWA results for color zoomed in on chr8. The locations of three inversions (one in *Pattern* and two in *Mel-Stripe2*) are shown with green shading (see Fig. 8 for details based on comparative alignments of chr8 in different genomes).

**Fig. 8.**
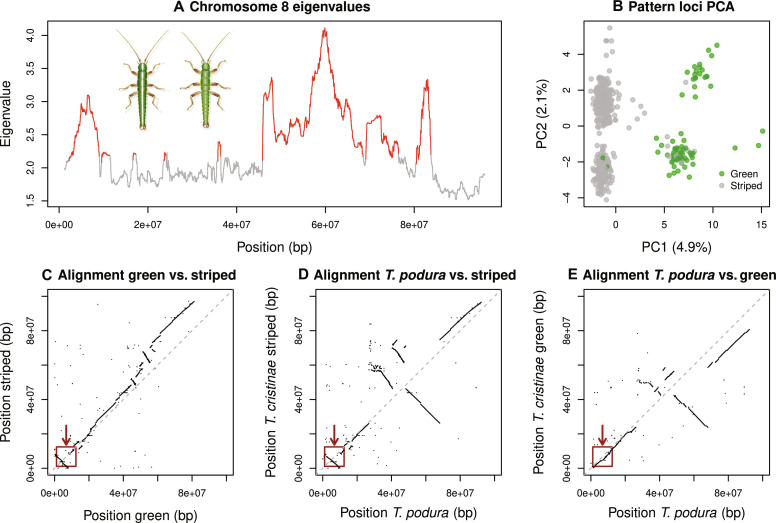
Genomics basis of the stripe and evidence for different genetic architectures in *T. cristinae* versus *T. podura*. (**A**) Principal components analysis (PCA) eigenvalues along chr8, which were used to delimit the peaks of association via a hidden Markov model approach. The two peaks, shown in red, delimit the *Pattern* locus (left) and the *Mel-Stripe2* locus (right). (**B**) PCA clusters based on genetic variation at the *Pattern* and *Mel*-*Stripe2* loci. (**C** to **E**) Comparative alignments that each compare two different chromosome-level assemblies, focused on chr8. The red box in the bottom-left corner highlights the *Pattern* locus. This shows how the highlighted region is similar between *T. podura* and the green morph *T. cristinae* genome, but rearranged between *T. podura* and the striped morph *T. cristinae* genome. Thus, *T. podura* appears to lack certain structural mutations associated with the striped morph in *T. cristinae*.

We used GWA analyses and comparisons of multiple chromosome-level genome assemblies to consider genomic variation underlying the stripe phenotype (Figs. 7 and 8 and fig. S6). GWA mapping of pattern within *T. cristinae* revealed that two large regions of chr8 associated strongly with color pattern, which we delimited as the *Pattern* locus [a 11.3–mega–base pair (Mbp) locus spanning 1,096,929 to 12,418,814 base pairs (bp)] and the *Mel-Stripe2* locus (a 39.2-Mbp locus spanning 45,229,604 to 84,397,122 bp) using a hidden Markov model approach (Figs. 7 and 8 and fig. S7).

We emphasize that the *Pattern* locus is reported here for the first time and its discovery here provides the first hints that color pattern is controlled by a single chromosome but, unlike implications from past work, involves more than a single locus. Moreover, *Mel-Stripe2* overlaps with the previously defined color locus, *Mel-Stripe* (a 10.5-Mbp locus originally defined based on the more fragmented melanic *T. cristinae* gnome), but (i) extends beyond the genomic region associated with color (Fig. 7D; based on a GWA using the stripe genome) and (ii) includes multiple peaks of association with pattern (Fig. 7D). Comparative alignments of green versus striped genomes in *T. cristinae* show that these two loci coincide with chromosomal inversions and other complex structural variants (Fig. 8). For example, *Pattern* corresponds almost exactly with a large (~12 Mbp) inversion, and *Mel-Stripe2* contains at least two moderately large inversions (~4 Mbp each).

We tested for excess heterozygosity for the newly defined *Pattern* and *Mel-Stripe2* loci, as predicted by the hypothesis that associative overdominance causes linked selection on color pattern and thereby contributes to the ongoing fluctuations in stripe frequency (i.e., that this is a source of selection that prevents NFDS from driving the stripe to a stable equilibrium frequency such that ongoing fluctuations are observed). A principal components analysis (PCA) of genetic variation in *Pattern* and *Mel-Stripe2* revealed six genetic clusters (fig. S8), as expected given the partially overlapping structural variation affecting color pattern (green versus stripe) and color (green versus melanic) (*55*). Treating these clusters as structural variant genotypes [as in (*47*)], we found a slight but nonsignificant excess of heterozygotes. Specifically, 57 of the 602 individuals were heterozygous, versus 49 expected by chance (null from 1000 binomial samples, *P* = 0.135). Thus, we did not detect clear evidence for (associative) overdominance.

These results suggest that the color pattern is likely determined by more than one locus on chr8, but, at the same time, imply that sufficiently few loci are involved that individual mutations matter for the evolution of this trait (Fig. 2). Thus, the stripe trait may be somewhat difficult to evolve and depend on the specific mutations (and complex structural variation specifically), an idea that we evaluate further below.

### Repeatability of crypsis at larger phylogenetic and temporal scales

**Fig. 9.**
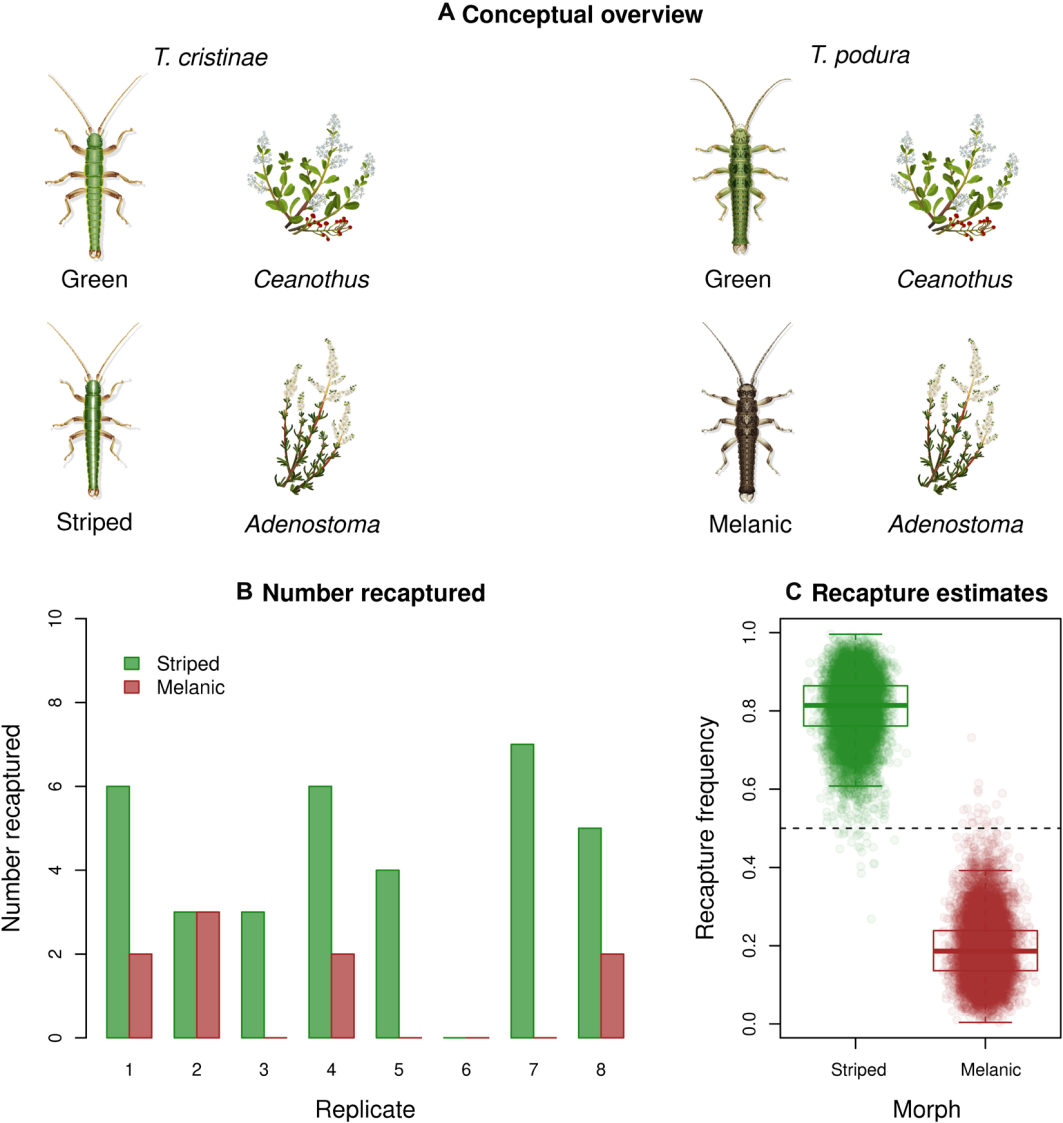
Two solutions to the problem of crypsis on *Adenostoma* (in *T. cristinae* versus *T. podura*) and an experimental test of which solution may offer higher fitness. (**A**) Conceptual overview of known patterns of crypsis. Both *T. cristinae* and *T. podura* use *Ceanothus* and *Adenostoma* as host plants and both exhibit a green unstriped morph that is cryptic on *Ceanothus*. In contrast, the two *Timema* species have evolved different morphs that are cryptic on *Adenostoma*, the striped morph in *T. cristinae* (left) and a melanistic gray/brown morph in *T. podura* (right). (**B**) Block-specific results of the mark-recapture field experiment testing the fitness of striped versus melanistic morphs of *T. cristinae* on *Adenostoma*. Shown is the number of each morph recaptured in each block (i.e., replicate), demonstrating higher recapture and thus fitness of the striped morph. (**C**) Average fitness of each morph across the entire experiment with the posterior distributions summarized in box plots (boxes denote the median, first and third quartiles of the posterior with whiskers extending to the minimum and maximum or 1.5 times the interquartile range) and individual parameter values sampled from the posterior overlain as points.

Although our time-series data are appreciable, they are, nonetheless, unlikely to capture rare events that occur at longer time intervals. Climatic events at the decadal scale have been implicated in evolutionary studies such as those of the famous Darwin’s finches (*20*). What about even longer timescales, beyond that of one or several human life spans? Two species of *Timema* provide insight into this question, in the context of the genetic architecture described above. Specifically, *T. cristinae* and *Timema podura* diverged millions of years ago and have evolved different solutions to the problem of crypsis on *Adenostoma* (*56*, *70*, *71*). Both *Timema* species use *Ceanothus* and *Adenostoma* as host plants and both exhibit a green unstriped morph that is cryptic on *Ceanothus*. In contrast, the two *Timema* species have evolved different morphs on *Adenostoma*, the afore-studied striped morph in *T. cristinae* and a melanistic gray/brown morph in *T. podura* (Fig. 9). Geographic variation and predation experiments in *T. podura* have shown that melanism increases fitness on *Adenostoma* relative to green coloration (*71*). Thus, the stripe phenotype is not a repeatable phenotypic outcome of living on *Adenostoma* at this phylogenetic scale.

Two core explanations come to mind for the lack of repeatability, one ecological and the second genetic, and the two hypotheses may act in conjunction. First, the use of different solutions could involve microenvironmental differences between the *Timema* species that exist despite the use of the same host genera (*56*, *70*, *71*). Environmental differences are likely given that the *Timema* species are found hundreds of kilometers apart, in mid- versus southern California, associated with chaparral versus more sub-alpine habitats. This “microenvironmental differences” hypothesis awaits tests in the future.

Second, *T. podura* may use the solution of melanism because mutations generating a stripe have not arisen and established in this species. A mark-recapture experiment within *T. cristinae* revealed that the stripe pattern has higher fitness on *Adenostoma* than does melanic coloration (Fig. 9, Bayesian generalized linear model, regression coefficient for the effect of stripe, β = 1.49, 95% ETPI = 0.58 to 2.79). This raises the possibility that stripe is a more optimal solution to crypsis on *Adenostoma* than melanism, implying that the contingency of mutation affects the color patterns of *Timema* observed in the wild (*4*). In other words, the evolution of a stripe may depend on specific mutations that make the repeated evolution of this trait challenging.

To further evaluate this idea, we generated a de novo, chromosome-level assembly for a green *T. podura* and compared it to the genomes from *T. cristinae*. This revealed that *T. podura* lacks at least one of the large structural variants (i.e., at the *Pattern* locus) that are associated with the stripe phenotype in *T. cristinae* (Figs. 7 and 8 and fig. S9). Specifically, at the *Pattern* locus, *T. podura* is collinear with respect to the green *T. cristinae* genome but inverted relative to the striped *T. cristinae* genome. Moreover, the *T. podura and* striped *T. cristinae* genomes differ by two large inversions within *Mel-Stripe2*, which do not appear to differ (or at least to the same extent) in the alignment of *T. podura* versus the green *T. cristinae* genome. Thus, different mutations likely control crypsis in the two species, with the mutations necessary for stripe only arising and persisting in *T. cristinae*. Together, our results imply that evolution is both repeatable and complex for the same trait (cryptic coloration), dependent on ecology, timescale, and mutation.

## DISCUSSION

**Fig. 10.**
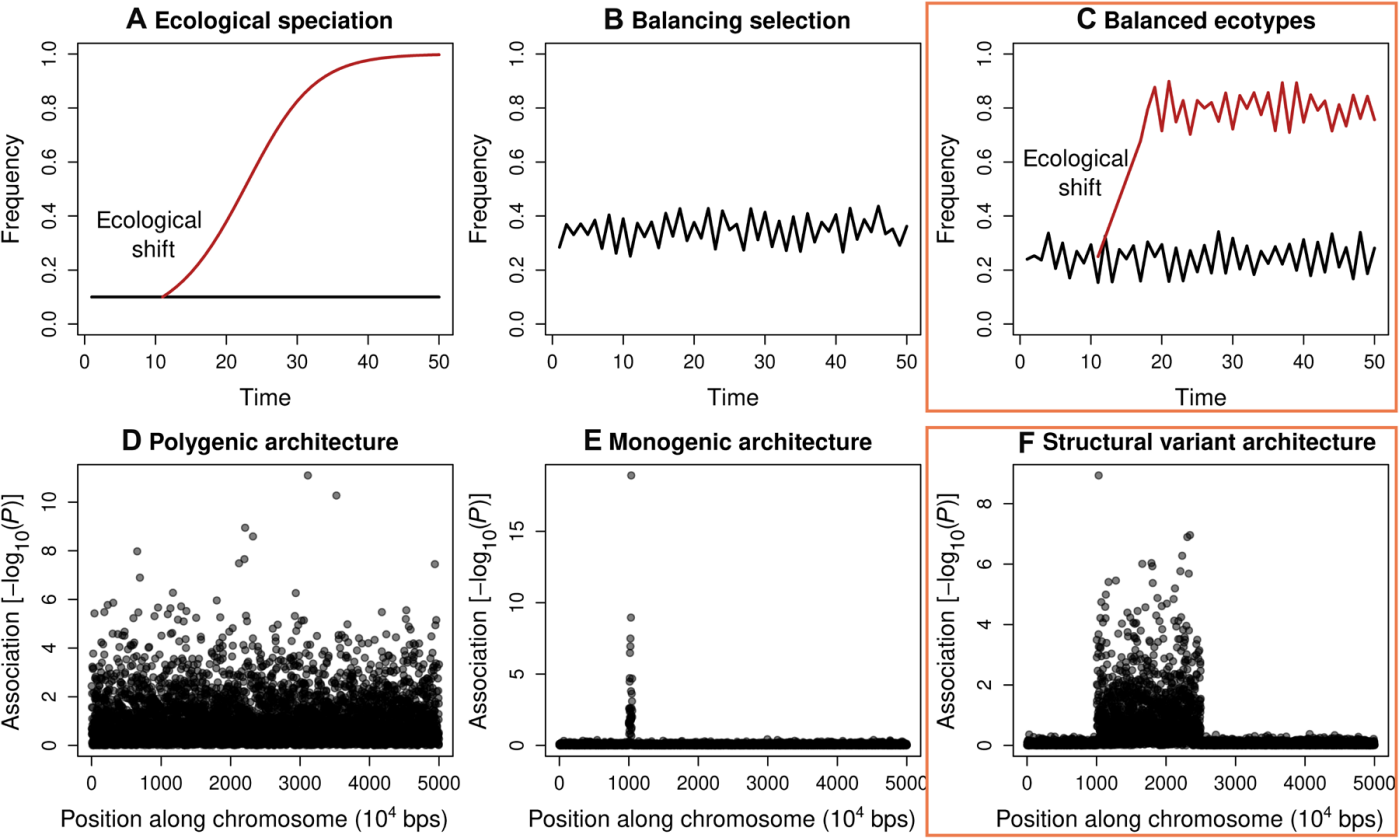
A schematic representation of the relationship between balanced polymorphism and ecological speciation. (**A**) The classic view of ecological speciation, where strong differentiation occurs between populations due to divergent selection between environments (here represented as the colonization of a new habitat by an ecological shift). (**B**) The classic view of balanced polymorphism where divergence does not occur and there are fluctuations within a single population. (**C**) A combination of the processes shown in (A) and (B), where there is some differentiation between populations, but it is not as strong as in (A) and fluctuations persist within each population such that the situation represents balanced ecotypes. The genetic architecture involved in scenarios (A) to (C) can also vary, depending on the type and number of traits under selection, thereby ranging from many loci (**D**), a major locus (**E**), or structural variation (**F**). The scenario observed in *T. cristinae* corresponds most closely to that shown in (C) and (F) (orange boxes for emphasis) but with the understanding that shown here are simple, stylized extremes for illustrative purposes.

We here reported notably repeatable changes in morph frequencies over time, in replicate long-term studies in the wild. Moreover, we showed that these fluctuations appear sustained, likely due to a combination of NFDS and other processes. In this context, the fact that color pattern is associated with structural variants that may be subject to other sources of selection (non-NFDS, e.g., linked selection) could be the very explanation for the sustained frequency fluctuations, rather than a confounding factor. Our results expand previous studies based on fewer populations and are particularly complementary to replicated studies of experimental evolution in the lab, albeit at much shorter timescales. We discuss our results in the context of timescale, the role of genetic variation in the repeatability of evolution, and the potential continuum between balanced polymorphism and speciation (Fig. 10). We acknowledge that our specific results pertain to the *Timema* system. Thus, we speculate below on how the results may differ or be similar in other taxa, thereby making predictions concerning generality.

### Repeatability across timescales

Like findings in microbes, our results illustrate how rather than being driven by selection “or” mutation, evolutionary dynamics reflect a context-dependent interplay between evolutionary factors (*72*, *73*). A key factor is whether the timescales involved allow for the emergence of new mutations (*2*, *31*). In this context, our combined time-series and genomic data provide a window into the link between evolution at short versus longer timescales (i.e., micro- versus macroevolution). Evolution from standing variation is often repeatable, as exemplified by lateral-plate evolution in stickleback fish (*2*). However, mutation introduces complexity at the longer timescales associated with Gould’s famous metaphor, also exemplified in stickleback fish in the context of the evolution of defensive spines (*74*, *75*). Thus, patterns of evolution are best understood through the effects of multiple sources of selection and genetic details concerning the mutations that affect adaptive traits.

### Genetic architecture and the repeatability of evolution

Because the genetic architecture of traits subject to selection can affect the repeatability of evolution, we quantified the genetic basis of color-pattern variation in *T. cristinae* and compared the genomes of *T. cristinae* and *T. podura*. We found that color pattern is associated with multiple genetic regions and structural variants on a single chromosome but a modest number of them, including the *Pattern* locus discovered and described in the current study. This expands past work which implied an even simpler (i.e., single locus) genetic basis to this trait but confirms a simple enough (i.e., not highly polygenic) basis such that individual mutations likely affect the dynamics of evolution. The finding of multiple loci implies that the sequence in which mutations arose at these loci could have affected evolutionary dynamics, as observed in studies of protein evolution (*4*, *36*, *38*). Future work should refine details of the complex structural variation associated with color pattern and potential interactions between loci, a task that will be facilitated by using even higher quality and phased genome assemblies. Such future work could then (i) date the origin of different structural variants to reveal whether and how they evolved in a stepwise fashion such that mutation sequence likely affected evolution (*76*) and (ii) attempt to infer how often adaptive mutations arise. Such insights could inform the extent to which mutations being rare versus path-dependent shape the repeatability of evolution, as studied in microbes (*34*).

Moreover, we note that body color (green versus melanic coloration) is associated with structural variants, but the frequencies of these two morphs do not fluctuate consistently and show no evidence of NFDS (*45*). Thus, fluctuations are not an inevitable outcome of polymorphism controlled by structural variation, and we suspect that multiple process may often contribute to evolutionary fluctuations, in our system and likely others (*58*). Further studies of genomic variation and natural selection in the wild, in *Timema* and other organisms, will likely continue to shed light on the role of genetic architecture in the repeatability of evolution (*33*).

### Relating balanced polymorphism to ecological speciation

Recent studies in *Timema* have focused on fluctuating natural selection and the maintenance of polymorphism (*40*, *47*), as did classic work on the system in the 1990s (*43*, *46*). However, a body of older work through the early 2000s focused on *Timema* as a model for the study of ecological speciation (*44*, *53*, *64*), i.e., the evolution of reproductive isolation (RI hereafter) as a consequence of divergent natural selection between environments (*54*, *77*–*79*). In the case of *Timema*, the different environments were presumed to be different host plant species. Several forms of RI were found to be greater between pairs of populations on different host plant species than between pairs of populations of similar genetic distance, which use the same host (*41*), a core prediction of ecological speciation (*77*). These forms of RI included “immigrant inviability” (*80*), due to natural selection against non-cryptic migrants (e.g., a striped individual moving onto *Ceanothus* may get eaten before genes are exchanged with largely green residents) (*64*), but also more traditional forms of RI such as sexual mating isolation (*44*, *53*), host preference (*81*, *82*), and cryptic postmating isolation (*42*). Thus, populations on different hosts form partially reproductively isolated “ecotypes,” consistent with an element of the process of ecological speciation but not its completion (*41*).

At face value, these bodies of work on polymorphism versus speciation may seem at odds with one another, the former concerns maintaining variation within populations and the latter differentially partitioning variation between populations, which can result in the loss of polymorphism. However, we note here that elements of both balancing selection and ecological speciation can operate simultaneously in the same system, essentially leading to “balanced ecotypes” (Fig. 10). In this situation, there is some differentiation between populations due to divergent selection between hosts. However, differentiation is counteracted by forms of balancing selection such as NFDS and gene flow. The end result is fluctuations around different equilibria under a balance between different sources of selection and gene flow, as observed in *T. cristinae*. The situation in *T. cristinae* may particularly represent ecotypes rather than strictly “morphs” because chr8 does not obviously control traits other than color and pattern, yet populations on different hosts do differ in a suite of other morphological, chemical, and behavioral traits (*41*, *42*, *56*, *81*, *82*).

We suspect such scenarios of balanced ecotypes may be common in organisms that live in highly heterogeneous environments that impose different forms of spatially and temporally varying selection, coupled with migration and gene flow. In less heterogeneous environments, perhaps ecological speciation proceeds to complete RI and loss of variation or the maintenance of polymorphism around a single equilibrium. While further research on the interplay between balancing selection, divergent selection, and gene flow is warranted, our results from *Timema* point to the potential plurality of process. In other words, the existence of one process or dynamic is not exclusive to the operation of others. Moreover, the genetic architecture underlying morphs, ecotypes, and species can differ in nonexclusive ways such that there is not a one-to-one relationship between genetics and the type of differentiation observed (Fig. 10).

### Predictability and repeatability in complex systems

In conclusion, we report here highly repeatable evolutionary fluctuations through time using replicated long-term field studies at the scale of several decades. We further demonstrate that the persistence of these fluctuations likely involves a complex interplay between NFDS and other processes, with mutation adding further complexity at longer timescales. Thus, the effects of NFDS are nuanced, yet this nuance can be critical for understanding and predicting evolution (*58*). In this context, our findings show that complexity and interaction can be of the essence for understanding biological diversity. This raises the possibility that further studies of the repeatability of evolutionary interactions could also provide insight into the dynamics of complex systems more generally (*83*, *84*), across the life, physical, and social sciences (*84*, *85*).

## MATERIALS AND METHODS

### Long-term field studies in *T. cristinae*

We compiled data on morph frequencies in *T. cristinae* using samples collected in the spring using sweep nets between 1990 and 2023. This builds on a previously published dataset that spanned the years 1990 to 2017 (*45*). All individuals were scored as “striped,” “green” (a green morph lacking a stripe), or “melanistic.” These classifications have been found to be highly repeatable in past work (*46*, *64*). Samples from 1990 to 1999 were collected and scored by Cristina Sandoval, who then trained PN in 2000. PN collected and scored most samples from 2000 to 2023. The host plant collected on (*Adenostoma* or *Ceanothus*) was recorded for all individuals. Detailed information on the collection localities (i.e., GPS coordinates and elevations), morph frequencies, sample sizes, etc., is provided in data S1.

We used this dataset to estimate the average frequency of the striped morph on *Adenostoma* and *Ceanothus* host plants each year of the time series. In this analysis, we analyzed the 32,867 stick insect observations from Santa Ynez mountain along highway 154 (i.e., we excluded a second mountain, Refugio mountain, from analysis but included these data in the data archive for completeness). This includes counts of stick insects from 213 population-by-year combinations on *Adenostoma* and 206 population-by-year combinations on *Ceanothus*. Here, we define a population as the stick insects collected from a locality on a specific host plant species. Treating the stick insects from different host plant species at the same locality as different populations is necessary to estimate morph frequencies by host. However, sympatric and parapatric host-associated populations, which are populations on different hosts but in the same or nearly the same spatial location, exhibit very low levels of genetic differentiation and modest differences in morph frequency (*45*, *46*, *86*). Thus, the analyses of frequency-dependent selection in subsequent sections focus on locality level data (i.e., pooled by host at a given locality) rather than distinguishing between host-associated populations at the same locality. Our analysis was conducted using a hierarchical Bayesian generalized linear model. See the “Hierarchical model for long-term field studies” section in the Supplementary Materials for details.

### Estimating *D* from the time-series data

The parameter *D* can be used to make inferences on selection, including NFDS. Specifically, theory shows that evolutionary dynamics and outcomes (e.g., stable oscillations and dampening oscillations) can be characterized by *D* (*57*, *58*), defined as

$$D={\left.\frac{\partial \Delta p}{\partial p}\right|}_{p=\hat{p}}$$

Here, *D* is the slope of the best fit line from the linear regression of Δ*p* (the change in frequency) on *p* (frequency) evaluated near the equilibrium (i.e., where Δ*p* = 0). Ignoring all other processes, gradual convergence to a stable equilibrium, $p=\hat{p}$, occurs when −1 < *D* < 0, dampened oscillations occur for −2 < *D* < −1, stable oscillations occur for *D* = −2, and diverging oscillations occur for *D* < −2 (*57*). *D* > 0 indicates positive frequency-dependent selection. Additional processes, such as genetic drift or fluctuating selection in a heterogeneous environment, can alter dynamics and outcomes relative to these expectations (*58*).

We thus sought to characterize NFDS dynamics and expected outcomes from our stripe-frequency time series by estimating *D*. We specifically estimated *D* for each of the 10 *T. cristinae* locations with 10 or more pairs of consecutive years (i.e., pairs of *p* and Δ*p*) (see table S1 for details about the 10 locations). We did this using a hierarchical Bayesian model, which accounted for uncertainty in stripe frequency in each location and year. See the “Hierarchical model for estimating *D* from the time-series data” in the Supplementary Materials for details.

We then estimated the expected equilibrium stripe frequency in each location as $p=\hat{p}$ = −α/*D*, where α is intercept term for the linear model for *D*. We computed the Pearson correlation coefficient to measure the association between the predicted equilibrium stripe frequency and the mean stripe frequency in each location. Last, we used linear regression to test for an association between the percentage of *Adenostoma* (versus *Ceanothus*) at each location and the predicted equilibrium stripe frequency. Host plant data were taken from Bolnick and Nosil (*60*) and, for one population (FHA), from recent observations by PN and (*83*). All analyses described in this section were conducted using R version 4.2.2.

### Field experiment testing the form of the NFDS function

We collected hundreds of stick insects from several core localities to conduct a field transplant experiment and thereby estimate the NFDS fitness function. A total of 513 individuals were collected from locations LA (latitude, 34.41°N; longitude, 119.80°W), PRC (latitude, 34.53°N; longitude, 119.86°W), PRNC (latitude, 34.53°N; longitude, 119.85°W), VPC (latitude, 34.53°N; longitude, 119.85°W), and WTA (latitude, 34.52°N; longitude, 119.78°W) between 30 April and 4 May 2021. Numbers were as follows: green morphs: LA, 17; PRC, 64: PRNC, 58: VPC, 71: and WTA, 48; striped morphs: LA, 136: PRC, 0; PRNC, 3; VPC, 26; and WTA, 90. Individuals were randomly assigned to one of 21 treatments (*n* = 20 stick insects per treatment), which varied the initial stripe frequency from 0% (0 striped, 20 green) to 100% (20 striped, 0 green) in 5% increments. Each of these treatments of 20 individuals was then randomly assigned to one of 21 experimental bushes (near locality N2, in the general area of latitude of 34.51°N and longitude of 119.80°W). Each bush was cleared of existing *T. cristinae* (the only *Timema* species occurring in this area) by sampling it before release. Past work demonstrates that this clears bushes of the overwhelming majority of *Timema* (*43*, *46*, *67*). Individuals were released on the morning of 5 May by dumping them from containers; the insects cling to the branches when released in this way because of a strong clinging reflex. Individuals were recaptured using visual surveys and sweep nets on the evening of 7 May, as in past work (*43*, *46*, *65*, *67*), and scored as striped or green. Past work suggests minimal dispersal occurs from the focal bush and that recapture is a good proxy for survival (*64*, *65*).

We fit a Bayesian model to test for NFDS in this experiment and quantify the relationship between stripe frequency and fitness. We considered two alternative models, specifying either a linear or sigmoidal (nonlinear, step-like) function for the relative fitness of the striped morph as a function of stripe frequency. Full details are provided in the “Model fitting for the field experiment testing the form of the NFDS function” section within the Supplementary Materials. These models allowed for flexible specification for the effect of stripe frequency on fitness; however, they did not give a direct estimate of *D*, the key NFDS parameter. Thus, we fit an additional model to estimate *D* in a manner analogous to the time-series data. This Bayesian model was identical to that described in the Supplementary Materials for estimating *D* from the time-series data (see the “Hierarchical model for estimating *D* from the time-series data” section in the Supplementary Materials), except that a single *D* was inferred (rather than one per locality), and, thus, the model was not hierarchical. Details of the priors and model fitting for this model are also provided in the Supplementary Materials (see the “Model fitting for the field experiment testing the form of the NFDS function” section).

### Testing for dampened oscillations and for the effect of drift on frequency fluctuations

If constant NFDS was the only evolutionary process operating, then the estimated values of *D* from the 10 *T. cristinae* localities would predict dampening oscillations in stripe frequency that gradually converge to a constant at the locality-specific equilibrium frequency (*57*, *58*). Thus, we first asked whether there was empirical evidence of such convergence in the time-series data. To do this, we fit a hierarchical Bayesian model for stripe frequency change (Δ*p*) as a function of time (year, which is equivalent to generation); we considered only the consistently sampled years from 2000 onward for this analysis. The hypothesis of dampening oscillations would predict a negative effect of year on the absolute value of stripe frequency change. The model and model fitting procedure are described in detail in the “Hierarchical Bayesian model testing for dampened oscillations” section in the Supplementary Materials.

As reported in Results, we found no evidence of a decline in the magnitude of frequency change over time, and, thus, some processes additional to NFDS alone are required to explain the persistent fluctuations in stripe, given the estimated values of *D* from our time series (*58*). One possibility is that NFDS combined with only genetic drift could explain the observed fluctuations. We used simulations of evolution by NFDS and drift to test this hypothesis. Specifically, for each location, we simulated replicate runs of evolution starting from the observed initial stripe frequency. We determined the expected stripe frequency each generation as Δ*p* = *D* (*p* − $p=\hat{p}$), where *D* and $p=\hat{p}$ are the estimated values of the NFDS parameter *D* and the estimated equilibrium stripe frequency for the relevant location from the hierarchical Bayesian time-series model, respectively (as reported in table S1). We incorporated genetic drift into each simulation by binomial sampling, such that the simulated stripe frequency after a generation of NFDS and drift was *p*′ ~ binomial(*p* + Δ*p*, *n* = 2*N*_e_). For this, we used a published estimate of contemporary *N*_e_ for the *T. cristinae* population FHA (latitude, 34.52°N; longitude, 119.80°W), based on the magnitude of genome-wide allele frequency change between two sampled generations, *N*_e_ = 110 (*45*, *66*). We conducted 100 replicate simulations for each of the 10 locations and summarized each simulation in terms of the mean absolute change in stripe frequency per generation. We then compared the observed mean absolute change from each of the 10 locations to the corresponding null distribution. As a sensitivity analysis, we repeated this entire procedure for values of *N*_e_ between 100 and 20 in increments of 10 (fig. S5). All analyses described in this section were conducted using R version 4.2.2.

### Reference genome for the green *T. cristinae* morph

In total, this study used three, independently assembled chromosome-level reference genomes, including an existing reference genome for the striped *T. cristinae* morph (*55*) and two previously unpublished reference genomes described for the first time in this paper (one for the green *T. cristinae* morph and one for *T. podura*). We created the de novo reference genome for the green *T. cristinae* morph using a combination of PacBio and Illumina reads from Chicago and Hi-C genomic libraries. DNA extraction, library preparation, DNA sequencing, and reference genome assembly was performed by Dovetail Genomics (completed June 2020). A single female stick insect from the locality Paradise Road North (PRCN; latitude, 34.53°N; longitude, 119.85°W; collected on *Ceanothus spinosus* in 2019) was used for the assembly, and the individual was chosen on the basis of a preliminary analysis that suggested it was homozygous for the green (unstriped) allele. See the “Reference genome DNA sequencing and assembly for the green *Timema cristinae* morph” section in the Supplementary Materials for a detailed description of the genome assembly procedure.

The final assembly comprised 1,313,928,163 bp with an N50 of 63,125,338 bp and an L50 of 7. The assembly included 13 large scaffolds corresponding to the 12 *T. cristinae* autosomes and the X chromosome (as described in more detail below) (fig. S6). On the basis of BUSCO version 4.0.5 (linage dataset, eukaryota_odb10; 70 species, 255 BUSCOs), the assembly included 195 complete BUSCOs (188 single copy and seven duplicated) and 10 fragmented BUSCOs; 50 BUSCOs were missing.

We then aligned this *T. cristinae* genome generated here to our existing chromosome-level reference genome for the striped *T. cristinae* morph (*55*, *72*). We did this using *Cactus* (version 1.0.0) (*87*, *88*). First, we used *RepeatMasker* (version 4.0.7) to mask repetitive regions of the genome (*89*) based on a repeat library developed for *Timema* (*55*). We then performed a pairwise alignment between the genomes with *Cactus* and used *HalSynteny* to extract syntenic alignment blocks from the comparative alignment (*90*). This was done with the default lower bound for synteny blocks of 5000 bp. We then identified homologous chromosomes by summing the total length of syntenic segments between the two genomes; there was a one-to-one correspondence between chromosomes. We then constructed sequence alignment dot plots from the synteny blocks using R (version 4.2.2). This allowed us to visualize inversions and other structural variation between the genomes of the two morphs. We focused on chr8 because of past evidence that it affects color pattern (results from our current study further support this) (*47*).

### GWA mapping of color pattern and color within *T. cristinae*

We used GWA mapping to identify regions of the genome associated with color pattern (striped versus green) and color (green versus melanic) variation in *T. cristinae*. For this, we used a previously published DNA sequence dataset comprising partial genome sequences (genotyping-by-sequencing data) for 602 *T. cristinae* from a single population (FHA; latitude, 34.52°N; longitude, 119.80°W) (*56*). We aligned these data to both the green genome generated here and the existing striped *T. cristinae* reference genome. This was done using the bwa aln algorithm (version 0.7.17) (*91*) with a maximum mismatch of 4 and allowing no more than 2 mismatches in the first 20 bp of each sequence. Bases with quality score less than 10 were removed and only reads with a single best alignment (-n 1) were kept. We use samtools (version 1.5) to compress, sort, and index the alignments. We then called single-nucleotide polymorphisms (SNPs) using samtools (version 1.5) and bcftools (version 1.6) (*92*). We used the consensus caller (-c), applied the recommended mapping quality adjustment for Illumina data (-C 50), and only output SNPs when the probability of all individuals being homozygous for the reference allele conditional on the data was <0.01. We used a series of Perl scripts to filter each variant set (i.e., based on each reference genome). These filters retained SNPs that met the following criteria: 2× minimum coverage per individual, a minimum of 10 reads supporting the non-reference allele, Mann-Whitney *P* values for base quality, mapping quality and read position rank sum tests > 0.005, a minimum ratio of variant confidence to non-reference read depth of 2, a minimum mapping quality of 30, no more than 20% of individuals with missing data, only two alleles observed, and coverage not exceeding 3 SDs of the mean coverage (at the SNP level). A total of 169,051 SNPs passed these filters and were used for GWA mapping based on the striped genome (121,635 for the green genome).

We used gemma to fit linear mixed models and thereby test for associations between each SNP and color pattern, measured quantitatively as percentage of the body area striped [the color-pattern data are described and reported in (*56*)]. This was done with the 538 stick insects that were green or striped (melanic stick insects were excluded). This method uses a kinship matrix to control for population genetic structure when testing for genotype-phenotype associations (all samples are from a single population, and thus limited structure is expected) (*93*). This analysis was performed using gemma version 0.95a with SNPs based on alignment and variant calling with the striped and green genomes. Likelihood ratio tests were used to compute *P* values for the null hypothesis test of no association between genotype and phenotype. We mapped color that is green (striped and unstriped) versus melanic, similarly, but with green coded as 0 and melanic coded as 1. This was done to compare GWA signals for color pattern to those of color.

### Determining the bounds of the color-pattern loci

The GWA mapping analysis identified two large blocks of SNP associations with color pattern on chr8 (see Fig. 7 and fig. S7). We hypothesized that these two large regions of association comprised regions of reduced recombination driven by structural genetic variation, as was previously suggested by past association mapping analyses of color and color pattern based on more fragmented genome assemblies (*45*, *47*). Regions of reduced recombination and structural variation can manifest as an increase in PCA eigenvalues for the first principal components (PCs) and clustering of individuals in PC space based on structural variant alleles [e.g., (*47*, *94*, *95*)]. We thus used genenticaly localized PCAs in combination with the GWA signal to delineate two large genetic regions (loci) associated with color pattern variation, denoted *Pattern* and *Mel-Stripe2*: *Pattern* = 1,096,929 to 12,418,814 bp, and *Mel-Stripe2* = 45,229,604 to 84,397,122 bp (see the “Methods for delimiting the bounds of the color-pattern loci” section in the Supplementary Materials for details). We examined the correspondence between these color-pattern loci and structural variation between the green and striped *T. cristinae* genomes as identified from the whole genome comparative alignment described above. This revealed a single large inversion corresponding approximately with *Pattern*, and several large inversions in *Mel-Stripe2* (along with other putative structural variation).

### Testing for an excess of heterozygotes at the color-pattern loci

We tested for an excess of heterozygous color-pattern genotypes in our focal FHA population (i.e., the same population used for GWA mapping). To do this, we first conducted a PCA of the genotypic data for SNPs within *Pattern* and *Mel-Stripe2* for the 602 stick insects (2020 SNPs total). We visually identified six genetic clusters in PC space (PCs 1 and 2), consistent with expectations for partially overlapping structural variants affecting color pattern and color (three clusters representing individuals homozygous for the green, striped, and melanic structural variant haplotypes, and three heterozygote clusters) (*47*). We thus used *k*-means clustering in R to assign individuals to the six genetic clusters (genotype groups) based on their PC1 and PC2 scores. This was done with 20 starts and a maximum of 500 iterations per start. We designated the genotype for each genetic cluster based on the associations of the clusters with color and color pattern. From this, we determined the sample frequency of each genotype and allele (green/green, 0.005; striped/striped, 0.272; melanic/melanic, 0.088; green/striped, 0.095; green/melanic, 0.038; and striped/melanic, 0.502). We then generated a null distribution for the expected number of green/striped heterozygotes under Hardy-Weinberg expectations by repeatedly (1000 times) drawing samples of 602 individuals from a binomial distribution with the probability of success equal to the expected heterozygote frequency (twice the product of the green and striped allele frequencies = 0.0815 = 2 * 0.0714 * 0.571).

### Experimental test of the selective advantage of the striped morph on *Adenostoma*

We conducted a second field transplant experiment to ask whether striped *T. cristinae* morphs exhibit higher fitness on *Adenostoma* than melanic morphs. For this experiment, stick insects were collected from a single location, PRNC (latitude, 34.53°N; longitude, 119.85°W). Stick insects for each pair of eight experimental blocks were captured on a single day in 2023: 9 May for blocks 1 and 2, 11 May for blocks 3 and 4, 13 May for blocks 5 and 6, and 15 May for blocks 7 and 8. For each block, 10 striped and 10 melanic stick insects (*n* = 20 total stick insects per block) were released onto a single *Adenostoma* bush at the experimental transplant locality (latitude, 34.51°N; longitude, 119.80°W; as in the first experiment) the day after they were collected. Despite past evidence for limited dispersal off experimental bushes (*64*, *65*), we explicitly tested this assumption again by marking the underbelly of the experimental stick insects with a fine-tip sharpie pen before release. Other than marking stick insects, the release procedures were as described for the NFDS experiment. Two days after release, surviving stick insects were recaptured using visual surveys and sweep nets [see, e.g., (*43*, *46*, *65*, *67*)] and scored as striped or melanic. Nearby bushes [within ~10 m, which is roughly the per-generation dispersal rate; (*52*)] were searched for marked *T. cristinae* stick insects to detect dispersal from the focal experimental *Adenostoma* bush. No marked stick insects were found off the focal bush in any of the eight blocks. We then fit a hierarchical Bayesian generalized linear model to determine whether striped *T. cristinae* were more fit than melanic morphs on *Adenostoma*. Details of the Bayesian model specification and fitting are provided in the Supplementary Materials (see the “Hierarchical Bayesian model testing the selective advantage of the striped morph on *Adenostoma*” section).

### Reference genome assembly for *T. podura* and comparative alignment with *T. cristinae*

We created a de novo reference genome for *T. podura* and report it here for the first time. This was done using a combination of PacBio and Illumina reads from an Omni-C genomic library. DNA extraction, library preparation, DNA sequencing, and reference genome assembly were performed by Dovetail Genomics (completed December 2021). A single female stick insect (green morph, ID = 17_0262) from Bay Springs, CA (latitude, 33.82°N; longitude, 116.70°W; collected on *Ceanothus* in 2017), was used for the assembly. See the “Reference genome DNA sequencing and assembly for *Timema podura*” section in the Supplementary Materials for details concerning sequencing and genome assembly.

The final assembly comprised 1,202,903,989 bp with an N50 of 73,318,109 bp and an L50 of 7. The assembly included 14 large scaffolds corresponding to the 12 *T. cristinae* autosomes and the X chromosome (fig. S9). On the basis of BUSCO version 4.0.5 (linage dataset, eukaryota_odb10; 70 species, 255 BUSCOs), the assembly included 233 complete BUSCOs (232 single copy and seven duplicated) and eight fragmented BUSCOs; 14 BUSCOs were missing.

Next, we aligned our *T. podura* genome to the our green morph *T. cristinae* genome (see above) and to the existing chromosome-level reference genome for the striped *T. cristinae* morph (*55*, *72*). We did this separately for each pair of genomes using Cactus (version 1.0.0), as described above for the *T. cristinae* comparative genome alignment (*87*, *88*). Briefly, this involved repeat masking with RepeatMasker (version 4.0.7) (*89*), pairwise genome alignment with Cactus, and the extraction of syntenic alignment blocks from the comparative alignment with HalSynteny (*90*). We then identified homologous chromosomes by summing the total length of syntenic segments between the two genomes. There was a one-to-one correspondence between most chromosomes, but *T. cristinae* chromosomes 1, 3, and 4 were each homologous with portions of *T. podura* scaffolds 3 and 12, and chromosome 3 and 4 also shared homology with *T. podura* scaffolds 2 (chromosome 3 only) and 8 (chromosome 4 only) (see fig. S9). We then constructed sequence alignment dot plots of *T. cristinae* chr8 from the synteny blocks using R (version 4.2.2) to visualize inversions and other structural variation between morphs (this chromosome corresponded with a single scaffold, number 29, in *T. podura*).

## Supplementary Materials

### This PDF file includes:

Materials and Methods Tables S1 to S4 Figs. S1 to S9 Legend for data S1 References
