# Supplementary material for: Evolution repeats itself in replicate long-term studies in the wild
Source: Sci Adv. 2024 May 24;10(21):eadl3149. doi: 10.1126/sciadv.adl3149 (PMC11122682; doi:10.1126/sciadv.adl3149)
Supplement: Supplementary file 1 — Materials and Methods Tables S1 to S4 Figs. S1 to S9 Legend for data S1 References [file sciadv.adl3149_sm.pdf]

Science Advances
AAAS

Supplementary Materials for

Evolution repeats itself in replicate long-term studies in the wild

Patrik Nosil *et al.*

Corresponding author: Patrik Nosil, patrik.nosil@cefe.cnrs.fr; Zach Gompert, zach.gompert@usu.edu

Sci. Adv. **10**, eadl3149 (2024)
DOI: 10.1126/sciadv.adl3149

The PDF file includes:

Materials and Methods
Tables S1 to S4
Figs. S1 to S9
Legend for data S1
References

Other Supplementary Material for this manuscript includes the following:

Data S1

Supplemental Materials and Methods

Hierarchical model for long-term field studies

We used data on morph frequencies in *T. cristinae* from 1990 to 2023 (32,867 stick insect observations, with 213 population by year combinations on *Adenostoma* and 206 population by year combinations on *Ceanothus*) to estimate the average frequency of striped morphs on *Adenostoma* and *Ceanothus* host plants each year of the time series. We did this using a hierarchical Bayesian generalized linear model. We assumed a binomial likelihood for the number of striped stick insects for each population and year, with a population (j) and year (i) specific probability of stripe (p_{ij}) and sample size (n_{ij}). Melanistic stick insects were excluded from this analysis. Thus, our estimate of stripe frequency is out of the total number of striped and green stick insects. We then defined a linear model for the logit frequency of stripe for each *Adenostoma* and *Ceanothus* population for each year as:

$$\text{logit}(p_{ij}) = \alpha_j + \beta_i$$

Here, α and β are population and year specific effects, where population refers to a specific location and host plant. We placed separate normal priors on the α parameters for *Adenostoma* and *Ceanothus* populations, with means (μ_A and μ_C) and precisions (i.e., reciprocal of the variance, τ_A and τ_C) estimated from the data. Separate normal priors were also placed on the year effects for *Adenostoma* and *Ceanothus* with means set to 0 and precisions estimated from the data. Sum-to-zero constraints were imposed on the year effects to ensure model identifiability. We then placed mostly uninformative priors on these higher level means and precisions, $\mu \sim \text{normal}(\text{mean} = 0, \text{precision} = 1e^{-6})$ and $\tau \sim \text{gamma}(\text{shape} = 0.01, \text{rate} = 0.001)$. Given the data sample size, these priors contain a trivial amount of information, e.g., the gamma priors denote a prior sample size of $2 * \text{shape}$ or 0.02 observations compared to the the 32,867 data observations, and thus the priors have a negligible effect on the posterior distributions.

We fit this model via Markov chain Monte Carlo (MCMC) using the `rjags` (version 4.14) interface with `JAGS` (98). We ran three chains each with a 10,000 iteration burn-in followed by 20,000 iterations for sampling with a thinning interval of 5. We verified likely convergence of the MCMC algorithm to the posterior distribution by computing Gelman and Rubin's potential scale reduction factor using the `gelman.diag` function from `coda` (version 0.19.4) (99). The upper bound of the 95% confidence interval for the potential scale reduction factor was less than 1.01 for all model parameters, consistent with convergence. Effective sample sizes for model parameters ranged from 495 to 12,696 (median = 9011, mean = 8257). This analysis was done using `R` version 4.2.2.

Hierarchical model for estimating D from the time-series data

We characterized NFDS dynamics and expected outcomes from our stripe-frequency time series by estimating D, defined as $D = \frac{\partial \Delta_p}{\partial p}\Big|_{p=\hat{p}}$. We specifically estimated D for each of the 10 *T. cristinae* locations with 10 or more pairs of consecutive years (i.e., pairs of p and Δp) (see Table S1 for details about the 10 locations). We did this using a hierarchical Bayesian model. We accounted for uncertainty in stripe frequency in each location (j) and year (i) by assuming the observed stripe count was binomially distributed, such that $y_{ij} \sim \text{binomial}(p_{ij}, n_{ij})$, where p_{ij} is the true (unobserved) stripe frequency and n_{ij} is the sample size. We then defined the following linear model:

$$\Delta p_{ij} = p_{i+1j} - p_{ij} = \alpha_j + D_j * p_{ij} + \epsilon_{ij}$$

Here, α_j is a location-specific intercept, D_j is the location-specific slope parameter of interest (D), and ϵ_{ij} is an error turn. We placed hierarchical priors on intercept and slope parameters, $\alpha_j \sim \text{normal}(\mu_\alpha, \sigma_\alpha)$ and $D_j \sim \text{normal}(\mu_D, \sigma_D)$. The hyperparameters were given the following weakly informative priors and inferred from the data: $\mu_\alpha \sim \text{normal}(\text{mean} = 0, \text{SD} = 20)$, $\mu_D \sim \text{normal}(\text{mean} = 0, \text{SD} = 20)$, $\sigma_\alpha \sim \text{gamma}(2, 0.1)$, and $\sigma_D \sim \text{gamma}(2, 0.1)$. We

assumed a normal prior on the error terms with a mean of 0 and a standard deviation estimated from the data; the latter was given the following prior, $\sigma \sim \text{gamma}(2, 0.1)$. We fit this model using Hamiltonian Monte Carlo (HMC) (100) via the `rstan` (version 2.21.8) interface with `Stan` (101). We used the No-U-Turn (NUTS) sampler (102) and ran four HMC chains each comprising a 1000 iteration warmup and 1000 additional sampling iterations. We computed and examined the Gelman–Rubin convergence diagnostic (i.e., the potential scale reduction factor) to verify adequate HMC mixing and likely convergence of the HMC algorithm to the posterior distribution (all estimates were less than 1.01). Effective sample sizes for model parameters ranged from 410 to 7258 (median = 3941, mean = 3787). We repeated the analysis using less informative priors to assess the sensitivity of the results to the priors selected. Specifically, we increased the standard deviations for the normal priors to 100 (versus 20) and changed the gamma shape parameters to 1 (instead of 2). This resulted in poorer mixing (lower effective sample sizes) but yielded very similar estimates of D (Pearson correlation for D between alternative model specifications = 0.99, 95% confidence interval = 0.96 to 1.00).

We assessed repeatability of evolutionary dynamics and outcomes across the 10 locations in terms of whether estimates of D were all consistent with NFDS ($D < 0$) and suggestive of a similar outcome (e.g., all $-1 < D < 0$ indicative of gradual convergence to an equilibrium), and based on the variability (e.g., standard deviation) of D across locations (a smaller standard deviation implies greater repeatability, see, e.g., (103)).

NFDS theory

Analytical theory for NFDS presented in the main text (Figure 5) is adapted from (104) . We assumed the relative fitnesses of A_1A_1 homozygotes, A_1A_2 heterozygotes, and A_2A_2 homozygotes depended on the genotype frequencies as follows: $w_{11} = 1 - s_b\gamma_{12} + s_b\gamma_{22}$ (for A_1A_1), $w_{12} = 1 - s\gamma_{12}$ (for A_1A_2), and $w_{22} = 1 - s_b\gamma_{12} + s_b\gamma_{11}$ (for A_2A_2). Here, s denotes the effect

of heterozygote frequency on the fitness of heterozygotes, s_b captures frequency-dependence for homozygotes (57), and the γ terms denote the genotype frequencies. Genotype frequencies after selection were calculated as $\gamma'_{ij} = \gamma_{ij} w_{ij}/\bar{w}$, where $\bar{w}$ is the mean fitness. Allele frequencies were calculated from genotype frequencies. Results in Figure 5 are based on s = 0.75, 1.2, 1.5, and 1 and s_b = 1.5, 2, 2.5, and 3. The key parameter D can be calculated from s and s_b as $D = \frac{3s_b - 2s}{s_b + 2s - 8}$ (57). Thus, the values of D for presented in Figure 5 are -0.60, -1.00, -1.80, and -2.33.

Model fitting for the field experiment testing the form of the NFDS function

We fit a Bayesian model to test for NFDS and quantify the relationship between stripe frequency and fitness in the *T. cristinae* NFDS experiment. For this, we assumed that the number of green and striped stick insects recaptured from an experimental treatment (i.e., a single bush with a unique initial stripe frequency) was binomially distributed with a morph and treatment-specific survival probability (i.e., absolute fitness), $y_i^{green} \sim \text{binomial}(w_i^{green}, n_i^{green})$ and $y_i^{striped} \sim \text{binomial}(w_i^{striped}, n_i^{striped})$. Here, i denotes the treatment index (i.e., the index for the initial stripe frequency) and w_i^{green} and $w_i^{striped}$ denote the absolute fitnesses of green and striped morphs in treatment i. We then defined a pair of linear equations that specify the absolute fitness and relative fitness of the stripe morph as a function of the initial stripe frequency,

$$\text{logit}(\bar{w}_i) = \mu_{\bar{w}} + \beta_{\bar{w}} p_i^{stripe}$$
$$\frac{w_i^{stripe}}{\bar{w}_i} = e^{\mu_\omega + \beta_\omega p_i^{stripe}}.$$

In these equations, the μ terms serve as intercepts and the β terms capture the effects of initial stripe frequency on average survival probability (average of the two absolute fitnesses) and relative fitness of the stripe, here $\frac{w_i^{striped}}{\bar{w}_i}$. From these equations, it follows that the absolute fitness of the green morph is $w_i^{green} = 2\bar{w}_i - w_i^{striped}$. We placed weakly informative normal priors

on the μ and β terms, all $\mathrm{normal}(\mathrm{mean} = 0, \mathrm{SD} = 10)$. We fit this model using HMC via the `rstan` (version 2.21.8) interface with `Stan` (101). For this, we used four HMC chains with the NUTS sampler, each comprising 4000 warmup iterations followed by 4000 sampling iterations, and set the target average proposal acceptance probability (`adapt_delta`) to 0.9 (the default value is 0.8). We computed and examined the Gelman–Rubin convergence diagnostic to verify adequate HMC mixing and likely convergence of the HMC algorithm to the posterior distribution (all estimates of the potential scale reduction factor were $\leq$1.01). Effective sample sizes for core model parameters (i.e., not transformed or derived parameters) ranged from 717 to 3299 (median = 1208, mean = 1479).

We next compared this model to an alternative model allowing for a non-linear relationship between stripe frequency and fitness. The model was identical to the linear model described above, except a sigmoid function was used to model relative fitness,

$$\frac{w_i^{stripe}}{\bar{w}_i} = \frac{L}{1 + e^{-1*k*(p_i^{stripe} - c)}}$$

Here, L denotes the maximum relative fitness, k specifies the steepness of the curve and c is the stripe frequency at which the relative fitness is halfway between its minimum and maximum value. Depending on the parameter values, this function can approximate a step function (very steep curves) or a near-linear function (very shallow curves). We placed weakly informative normal priors on L and k, each with a mean of 0 and SD of 10, and an uninformative beta prior on c (both shape parameters set to 1). The same HMC specifications were used for this second model and again the Gelman-Rubin diagnostic indicated likely convergence to the posterior (i.e., all estimates of the potential scale reduction factor were $\leq$1.01).

We used approximate leave-one-out cross validation to compare this model to the linear model described above. This was done with the `loo` function from `rstan` (105). As shown in Table S3 and Figure S4, the two models performed similarly with the linear model marginally

outperforming the sigmoid model. However, even more importantly, both models produced in near linear relationships between stripe frequency and fitness. In other words, the best sigmoidal model approximated a linear model not a step model and is quite similar to the linear model despite the different prior structure specified by the model.

We fit an additional model to estimate D in a manner analogous to the time-series data. This second Bayesian model was identical to that described above for estimating D from the time-series data, except that a single D was inferred (rather than one per locality) and thus the model was not hierarchical. As such, we used weakly informative priors on α (the intercept) and D, both $\mathrm{normal}(\mathrm{mean} = 0, \mathrm{SD} = 1)$. We likewise placed a weakly informative prior on the residual standard deviation, $\sigma \sim \mathrm{gamma}(2, 0.1)$. Here, we again used four HMC chains with the NUTS sampler; in this case, each chain comprised a 3000 iteration warmup followed by 20,000 iterations for sampling and `adapt_delta` was set to 0.95. Once again, we computed and examined the Gelman–Rubin convergence diagnostic to verify adequate HMC mixing and likely convergence of the HMC algorithm to the posterior distribution. All estimates of the potential scale reduction factor were 1.00, consistent with convergence; effective sample sizes for core model parameters ranged from 155 to 9437 (median = 719, mean = 1407). We further verified that moderate changes to the specified priors did not unduly affect our inference. Specifically, we increased the normal standard deviation parameters from one to five and decreased the gamma shape parameters from two to one (both specifying less constraining/informative priors) and obtained nearly identical estimates of D (0.76 versus 0.77) (this was despite a decrease in mixing indicated by reduced effective sample sizes with the alternative, more permissive priors).

The equilibrium stripe frequency for the experimental location was predicted from these results (with the original prior specifications) as $\hat{p} = \frac{-\alpha}{D}$. All analyses described in this section were conducted using `R` version 4.2.2.

Hierarchical Bayesian model testing for dampened oscillations

We fit a hierarchical Bayesian model for stripe frequency change (Δp_{ij}) as a function of time (year, which is equivalent to generation) using only the consistently sampled years from 2000 onward for this analysis. The hypothesis of dampening oscillations would predict a negative effect of year on the absolute value of stripe frequency change. We assumed a normal likelihood for stripe frequency change, specifically $\Delta p_{ij} \sim \text{normal}(\mu_{ij}, \sigma)$, with a linear model for the expected change as a function of time (i) and locality (j)

$$\mu_{ij} = \alpha_j + \beta_j x_i^{year}.$$

In this equation, α_j is a location-specific intercept and β_j denotes the location-specific effect of year on the absolute value of change. We placed hierarchical priors on the regression parameters, $\alpha_j \sim \text{normal}(\mu_\alpha, \sigma_\alpha)$ and $\beta_j \sim \text{normal}(\mu_\beta, \sigma_\beta)$. We then placed weakly informative priors on the hyperparameters and estimated these from the data: $\mu_\alpha \sim \text{normal}(\text{mean} = 0, \text{SD} = 1)$, $\mu_\beta \sim \text{normal}(\text{mean} = 0, \text{SD} = 1)$, $\sigma_\alpha \sim \text{gamma}(2, 0.1)$, and $\sigma_\beta \sim \text{gamma}(2, 0.1)$. We likewise placed a weakly informative prior on the residual standard deviation term, $\sigma \sim \text{gamma}(2, 0.1)$. We fit this model using HMC (100) via the `rstan` (version 2.21.8) interface with `Stan` (101). We used the NUTS sampler (102) and ran four HMC chains each comprising a 1000 iteration warmup and 1000 additional sampling iterations. We again computed and examined the Gelman–Rubin convergence diagnostic to verify adequate HMC mixing and likely convergence of the HMC algorithm to the posterior distribution (all estimates of the potential scale reduction factors were 1.00). Effective sample sizes for core model parameters ranged from 455 to 2279 (median = 737, mean = 855).

Reference genome DNA sequencing and assembly for the green *Timema cristinae* morph

As noted in the main text, a single female stick insect from the locality Paradise Road North (PRCN, latitude 34.53°N, longitude 119.85° W, collected on *Ceanothus spinosus* in 2019) was used for the assembly. The DNA sample was quantified using Qubit 2.0 Fluorometer (Life Technologies, Carlsbad, CA, USA). The PacBio SMRTbell library (∼20kb) for PacBio Sequel was constructed using SMRTbell Express Template Prep Kit 2.0 (PacBio, Menlo Park, CA, USA) using the manufacturer recommended protocol. The library was bound to polymerase using the Sequel II Binding Kit 2.0 (PacBio) and loaded onto PacBio Sequel II. Sequencing was performed on PacBio Sequel SMRT cells. An initial genome assembly was then performed using the `FALCON` 1.8.8 pipeline from Pacific Bioscience. The assembly was polished through PacBio's Arrow algorithm from SMRT Link 5.0.1, using the original raw-reads.

A Chicago library was prepared as described in (106). Briefly, ∼500ng of high molecular weight gDNA was reconstituted into chromatin in vitro and fixed with formaldehyde. Fixed chromatin was digested with DpnII, the 5' overhangs filled in with biotinylated nucleotides, and then free blunt ends were ligated. After ligation, crosslinks were reversed and the DNA purified from protein. Purified DNA was treated to remove biotin that was not internal to ligated fragments. The DNA was then sheared to ∼350 bp mean fragment size and sequencing libraries were generated using NEBNext Ultra enzymes and Illumina-compatible adapters. Biotin-containing fragments were isolated using streptavidin beads before PCR enrichment of each library. The libraries were sequenced on an Illumina HiSeq X to a target depth of 30X coverage.

A Dovetail Hi-C library was prepared in a similar manner as described previously (107). Briefly, for each library, chromatin was fixed in place with formaldehyde in the nucleus and then extracted. Fixed chromatin was digested with DpnII, the 5' overhangs filled in with biotinylated

nucleotides, and then free blunt ends were ligated. After ligation, crosslinks were reversed and the DNA purified from protein. Purified DNA was treated to remove biotin that was not internal to ligated fragments. The DNA was then sheared to ~350 bp mean fragment size and sequencing libraries were generated using NEBNext Ultra enzymes and Illumina-compatible adapters. Biotin-containing fragments were isolated using streptavidin beads before PCR enrichment of each library. The libraries were sequenced on an Illumina HiSeq X to a target depth of 30X coverage.

The PacBio *de novo* assembly, Chicago library reads, and Dovetail Hi-C library reads were used as input data for `HiRise`, a software pipeline designed specifically for using proximity ligation data to scaffold genome assemblies (106). An iterative analysis was conducted. First, Chicago library sequences were aligned to the draft input assembly using a modified `SNAP` read mapper (`http://snap.cs.berkeley.edu`). The separations of Chicago read pairs mapped within draft scaffolds were analyzed by `HiRise` to produce a likelihood model for genomic distance between read pairs, and the model was used to identify and break putative misjoins, to score prospective joins, and make joins above a threshold. After aligning and scaffolding Chicago data, Dovetail Hi-C library sequences were aligned and scaffolded following the same method.

Methods for delimiting the bounds of the color-pattern loci

As noted in the main text, we used local PCAs in combination with the GWA signal to delineate two large genetic regions (loci) associated with color pattern variation, denoted *Pattern* and *Mel-Stripe2*. We first performed separate (local) PCA ordinations of the genetic data (centered but not standardized genotype matrixes) for the 5890 SNPs on chromosome 8 in 100-SNP sliding windows (5791 windows total). We then summarized each PCA by the eigenvalue associated with the first eigenvector. Visual inspection of sliding window eigenvalues suggested two peaks

of high eigenvalues (accentuated structure) on chromosome 8 coinciding with the GWA signal.

We formally delimited the color-pattern loci using a Hidden Markov model (HMM) approach. Specifically, we fit a HMM to the eigenvalues using the `HiddenMarkov` package (version 1.8.13) in `R` (108). We modeled two hidden states, which we initialized with expected values equal to the 25th and 75th percentiles of the empirical eigenvalue distribution for chromosome 8. We assumed a normal distribution for the observed eigenvalues with standard deviations initialized at half the empirical standard deviation. We then estimated the hidden state means, standard deviations, and transitions between hidden states using the Baum-Welch algorithm (i.e., we set initial values for means and standard deviations but these were then refined with the Baum-Welch algorithm) (109). For this, we allowed a maximum of 500 iterations and set the tolerance to $1e^{-4}$. Using this procedure, we identified states corresponding to high (mean eigenvalue = 2.67, SD = 0.43) and low or background (mean eigenvalue = 1.87, SD = 0.14) population structure (i.e., high and low eigenvalues). We then used the Viterbi algorithm for decoding, that is for inferring the most likely hidden state for each 100 SNP window (110). This algorithm assigned 4072 SNP windows to the low (background) state and 1719 windows to the high state. The 1719 high state windows comprised 8 contiguous regions of the high state that broadly overlapped with the GWA signal. We combined neighboring high windows separated by narrow regions of the low HMM state to define the two color pattern loci corresponding to the GWA signals: *Pattern* = 1,096,929 to 12,418,814 bps, and *Mel-Stripe2* = 45,229,604 to 84,397,122 bps.

Hierarchical Bayesian model testing the selective advantage of the striped morph on *Adenostoma*

We fit a hierarchical Bayesian generalized linear model to determine whether striped *T. cristinae* were more fit than melanic morphs on *Adenostoma*. We assumed a binomial likelihood

for the number of striped stick insects recaptured for each block (j), $y_j \sim \text{binomial}(p_j, n_j)$. Here, p_j and n_j denote the block-specific probability that a recaptured stick insect was striped and the block-specific number of stick insects recaptured. We assumed $\text{logit}(p_j) = \beta + \alpha_j$, where β denotes the mean effect of being striped (relative to melanic) and α is a replicate-specific deviation from this mean. We modeled the α hierarchically with a zero-centered normal prior, $\alpha_j \sim \text{normal}(\text{mean} = 0, \text{SD} = \sigma)$, and placed weakly informative priors on β and σ, $\beta \sim \text{normal}(\text{mean} = 0, \text{SD} = 10)$ and $\sigma \sim \text{normal}(\text{mean} = 0, \text{SD} = 1)$. We again fit this model with HMC and the NUTS sampled via the `rstan` interface with `Stan`. We used four HMC chains, each comprising a 1000 iteration warmup followed by an additional 1000 sampling iterations. We again computed and examined the Gelman–Rubin convergence diagnostic to verify adequate HMC mixing and likely convergence of the HMC algorithm to the posterior distribution (all values of the potential scale reduction factor were $\leq$1.01, consistent with convergence); effective sample sizes for core model parameters ranged from 964 to 8661 (median = 3974, mean = 4038). We then focused on the posterior distribution of β, the stripe effect, for inference. This analysis was conducted with `R` version 4.2.2. Using more permissive (less constraining or informative) priors again had little effect on inference. Specifically, estimates of β were very similar (posteriors medians of 1.49 versus 1.58 for the original versus less constraining priors) and estimates of the replicate effects (α's) were highly correlated (Pearson correlation = 0.998, 95% confidence intervals = 0.989 to 1.00).

Reference genome DNA sequencing and assembly for *Timema podura*

As noted in the main text, we created a *de novo* reference genome for *T. podura* using a combination of PacBio and Illumina reads from a Omni-C genomic library (all performed by Dovetail Genomics). The DNA sample from a single female *T. podura* was quantified using Qubit 2.0 Fluorometer (Life Technologies, Carlsbad, CA, USA). The PacBio SMRTbell library ($\sim$20kb)

for PacBio Sequel was constructed using SMRTbell Express Template Prep Kit 2.0 (PacBio, Menlo Park, CA, USA) using the manufacturer recommended protocol. The library was bound to polymerase using the Sequel II Binding Kit 2.0 (PacBio) and loaded onto PacBio Sequel II). Sequencing was performed on PacBio Sequel II 8M SMRT cells.

`wtdbg2` (111) was then run to generate a primary assembly. Next, `blobtools` v1.1.1 (112) was used to identify potential contamination in the assembly based on `blast` (v2.9) results of the assembly against the NT database. A fraction of the scaffolds was identified as contaminant and were removed from the assembly. The filtered assembly was then used as an input to `purge_dups` v1.1.2 (113), and potential haplotypic duplications were removed from the assembly, resulting in the final assembly.

For the Dovetail Omni-C library, chromatin was fixed in place with formaldehyde in the nucleus. Fixed chromatin was digested with DNaseI and then extracted, chromatin ends were repaired and ligated to a biotinylated bridge adapter followed by proximity ligation of adapter containing ends. After proximity ligation, crosslinks were reversed and the DNA purified. Purified DNA was treated to remove biotin that was not internal to ligated fragments. Sequencing libraries were generated using NEBNext Ultra enzymes and Illumina-compatible adapters. Biotin-containing fragments were isolated using streptavidin beads before PCR enrichment of each library. The library was sequenced on an Illumina HiSeqX platform to produce ~30X sequence coverage.

The input PacBio *de novo* assembly and Dovetail Omni-C library reads were used as input data for `HiRise`, a software pipeline designed specifically for using proximity ligation data to scaffold genome assemblies (106). The Dovetail Omni-C library sequences were aligned to the draft input assembly using `bwa` (91). The separations of Dovetail Omni-C read pairs mapped within draft scaffolds were analyzed by `HiRise` to produce a likelihood model for genomic distance between read pairs, and the model was used to identify and break putative misjoins, to

score prospective joins, and make joins above a threshold.

Tables

Table S1: Characteristics of the 10 replicate locations studied over time. Years = number of consecutive year pairs analyzed, Observations = total number of observations (i.e., the sample size in terms of numbers of individuals), D = Bayesian point estimate (median) and 95% equal-tail probability intervals for D, $\hat{p}$ = predicted equilibrium stripe frequency.

Location	Latitude°	Longitude°	Years	Observations	D	$\hat{p}$
FH	34.5176	-119.8010	10	4531	-0.38 (-0.65, -0.13)	0.92
HV	34.4884	-119.7864	22	4580	-0.32 (-0.51, -0.11)	0.71
L	34.5089	-119.7956	15	4101	-0.48 (-0.81, -0.21)	0.89
M	34.5151	-119.7971	15	1543	-0.42 (-0.74, -0.14)	0.67
MBOX	34.5035	-119.8056	10	884	-0.38 (-.060, -0.12)	0.68
OG	34.5128	-119.7962	13	1659	-0.45 (-0.76, -0.18)	0.78
OUT	34.5318	-119.8435	14	1664	-0.70 (-0.96, -0.40)	0.43
PR	34.5331	-119.8574	17	2403	-0.61 (-1.04, -0.25)	0.02
SC	34.5226	-119.8318	12	776	-0.58 (-1.05, -0.24)	0.05
VP	34.5324	-119.8473	12	5255	-0.50 (-0.83, -0.22)	0.21

Table S2: Bayesian estimates of the slope parameters relating the change in stripe frequency to time. Point estimates and 95% equal-tail probability intervals (ETPIs) for each of the 10 focal locations are shown.

Location	Slope (95% ETPI)
FH	0.0001 (-0.0019, 0.0021)
HV	0.0004 (-0.0012, 0.0021)
L	-0.0006 (-0.0024, 0.0011)
M	0.0009 (-0.0009, 0.0026)
MBOX	0.0043 (0.0018, 0.0073)
OG	0.0011 (-0.0011, 0.0032)
OUT	0.0025 (0.0007, 0.0043)
PR	0.0003 (-0.0014, 0.0020)
SC	0.0006 (-0.0014, 0.0029)
VP	0.0009 (-0.0009, 0.0028)

Table S3: Comparison of linear and sigmoid functions for relative fitness in the negative frequency-dependent selection (NFDS) experiment based on approximate leave-one-out (LOO) cross validation. Estimates and standard errors (in parentheses) are shown for the Bayesian LOO estimate of the expected log pointwise predictive density ($\mathrm{ELPD_{LOO}}$), the effective number of parameters ($\mathrm{P_{LOO}}$), and the LOO information criterion (LOOIC). Smaller values of LOOIC denote better models in terms of expected predictive performance.

Model	$\mathrm{ELPD_{LOO}}$	$\mathrm{P_{LOO}}$	LOOIC
Linear	-70.9 (6.7)	5.6 (1.1)	141.8 (13.3)
Sigmoidal	-71.6 (7.0)	6.4 (1.3)	143.2 (14.1)

Table S4: Comparison of mean absolute stripe frequency change and null expectations with drift and negative frequency-dependent selection (NFDS) assuming an effective population size of 110. Results are shown for each of 10 locations. Mean absolute change, the ratio of mean change to the mean null expectation (X-fold change) and the P-value from the null hypothesis test that the observed change did not exceed expectations for drift and NFDS are given.

Location	Mean change	X-fold change	P-value
FH	0.027	1.32	0.03
HV	0.045	1.62	<0.01
L	0.032	1.60	<0.01
M	0.056	1.69	<0.01
MBOX	0.075	2.65	<0.01
OG	0.042	1.70	<0.01
OUT	0.080	2.33	<0.01
PR	0.021	2.48	<0.01
SC	0.022	1.61	<0.01
VP	0.058	2.25	<0.01

Figures

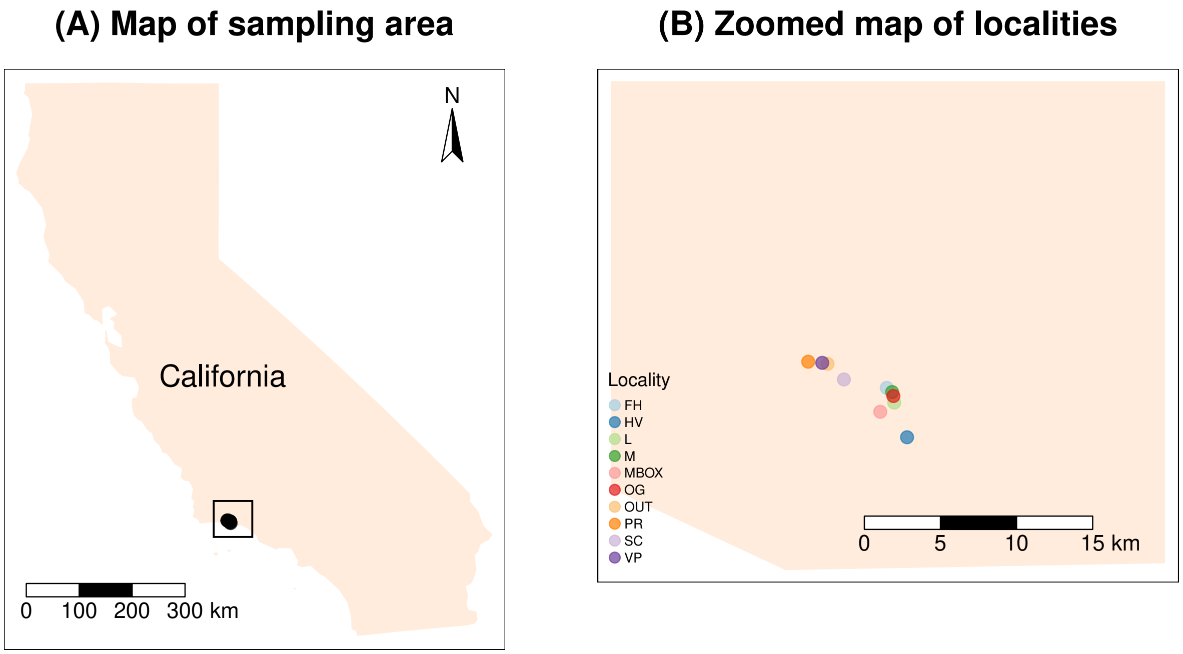

Figure S1: Map of *T. cristinae* localities. The maps show the 10 localities with long-term observational data (10 or more pairs of years). Panel (A) show the localities (black dots) in a larger geographic context, whereas panel (B) is zoomed in the specific geographic region within California where the localities exist (as indicated by the black box in panel A).

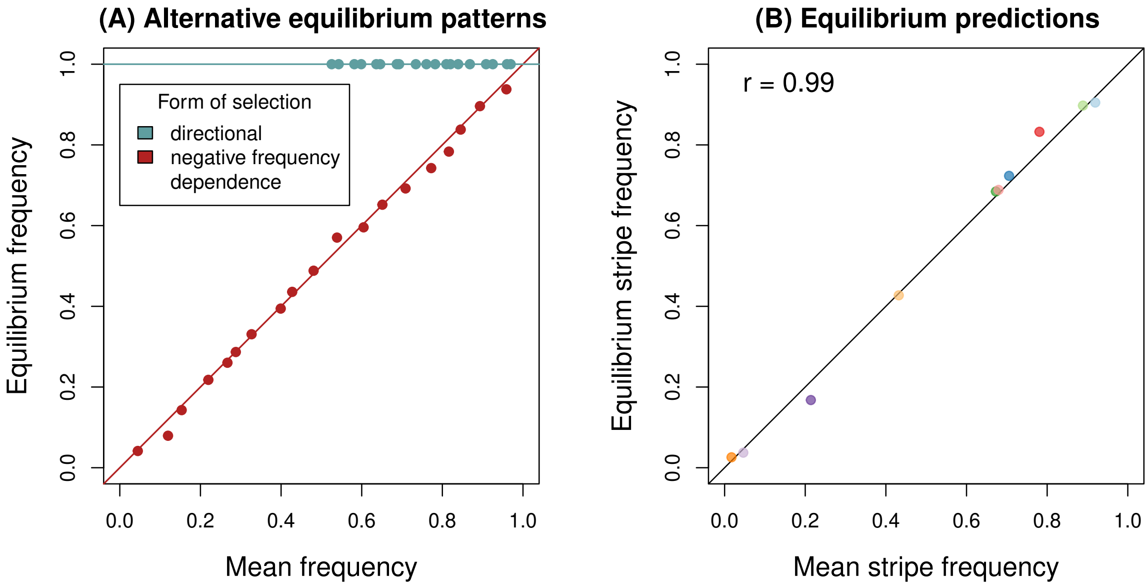

Figure S2: Replicate time series reveal a close match between observed and predicted equilibrium morph frequencies under negative frequency-dependent selection. Although the same data are used to estimate observed and predicted frequencies, the strong association detected in not a given and would not arise under all models of evolution (e.g., those with directional evolution rather than fluctuations due to NFDS). Panel (A) shows the general relationship between the mean frequency in a time series and the equilibrium predicted for directional selection favoring a morph or negative frequency-dependent selection. The relationship for the observed data is shown in (B) with points colored to denote locations as in Figure 1 of the main text (Pearson correlation = 0.99, $P < 0.0001$).

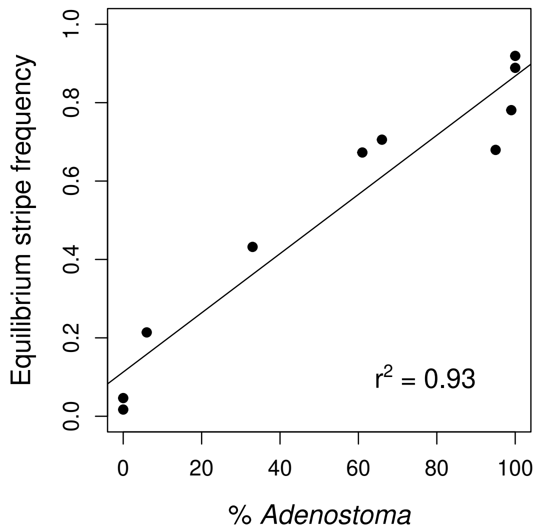

Figure S3: Predicted equilibrium stripe frequency as a function of the prevalence of *Adenostoma* at a locality. The scatterplot shows the percentage of *Adenostoma* (versus *Ceanothus*) and the predicted stripe frequency from the NFDS time series for each of the 10 focal sites. The best fit line from linear regression, which explained 93% of the variation in equilibrium stripe frequency ($P < 0.0001$), is shown.

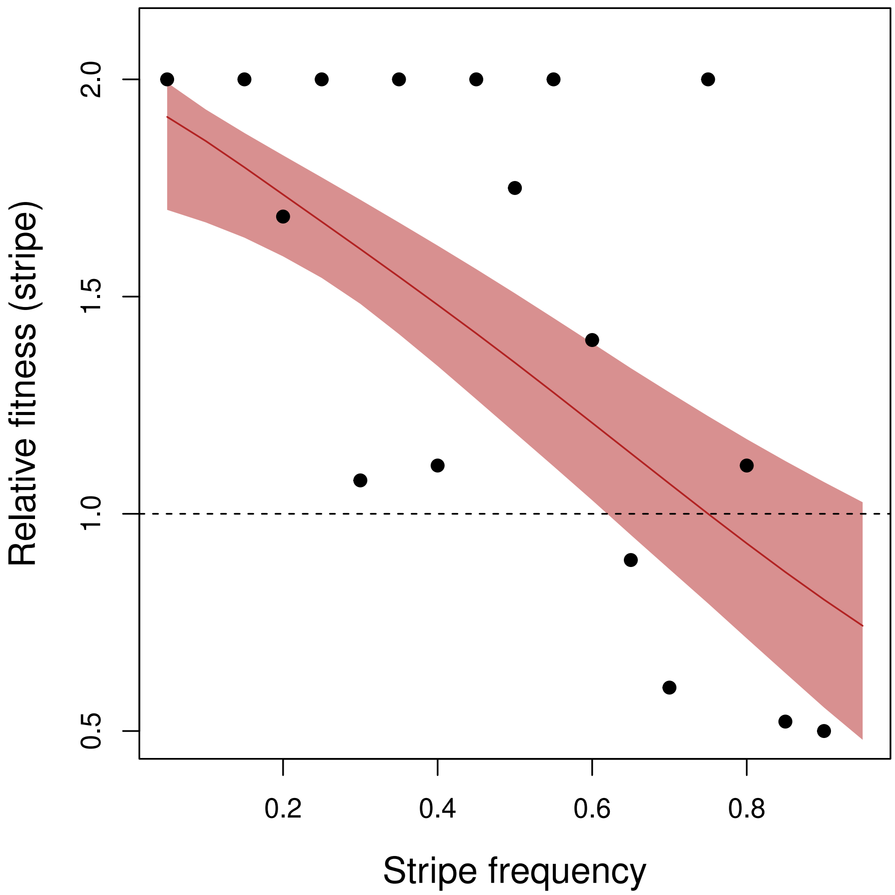

Figure S4: Relative fitness of striped versus green morphs as a function of stripe frequency. In contrast to the results shown in the main manuscript, this plot show results from fitting a sigmoid function for relative fitness (i.e., a non-linear model). Even with the assumed sigmoid model, the estimated parameters result in a relatively linear decline of relative fitness with frequency. Dots represent values for each experimental bush with lines and shading representing the estimated fitness function (medians and 95% equal-tailed probability interval, ETPI, respectively)

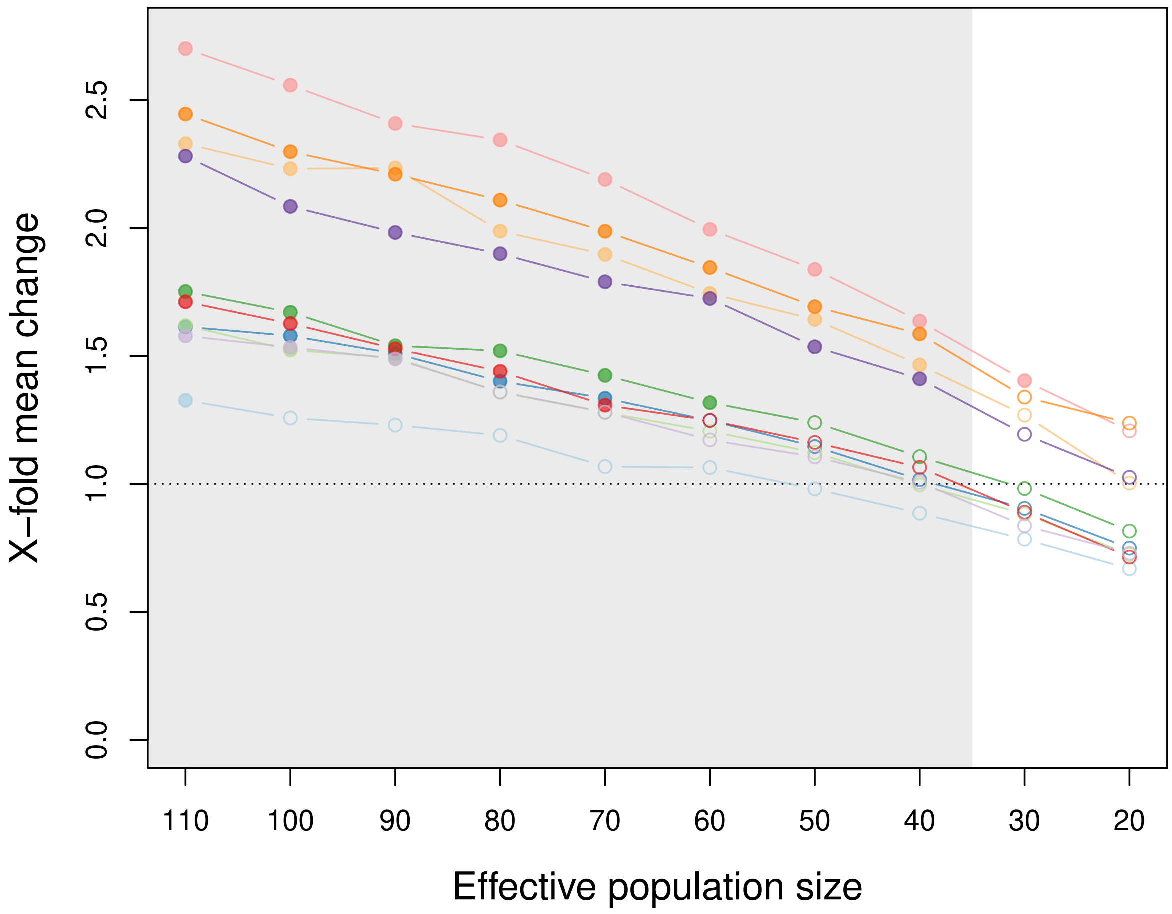

Figure S5: Graphical summary of the sensitivity analysis for explaining the observed fluctuations in stripe frequency by drift and negative frequency-dependent selection (NFDS). The plot shows the ratio of the mean absolute change in stripe frequency for each location relative to the expectation from simulations of evolution by drift (X-fold mean change). The dotted horizontal line indicates X-fold mean change of 1, which implies the observed mean change matches expectations for drift combined with NFDS. Results are shown for a range of effective population sizes, from 110 to 20. Colored points denote different locations (see the legend in Figure 1E). Filled circles denote conditions where the probability of the observed change by drift and NFDS was less than 0.05 (i.e., $P < 0.05$); open circles indicate $P \geq 0.05$. The shaded region of the plot encompasses effective population sizes where the Fisher combined P-value for the null hypothesis that drift can explain change for all 10 locations was less than 0.05.

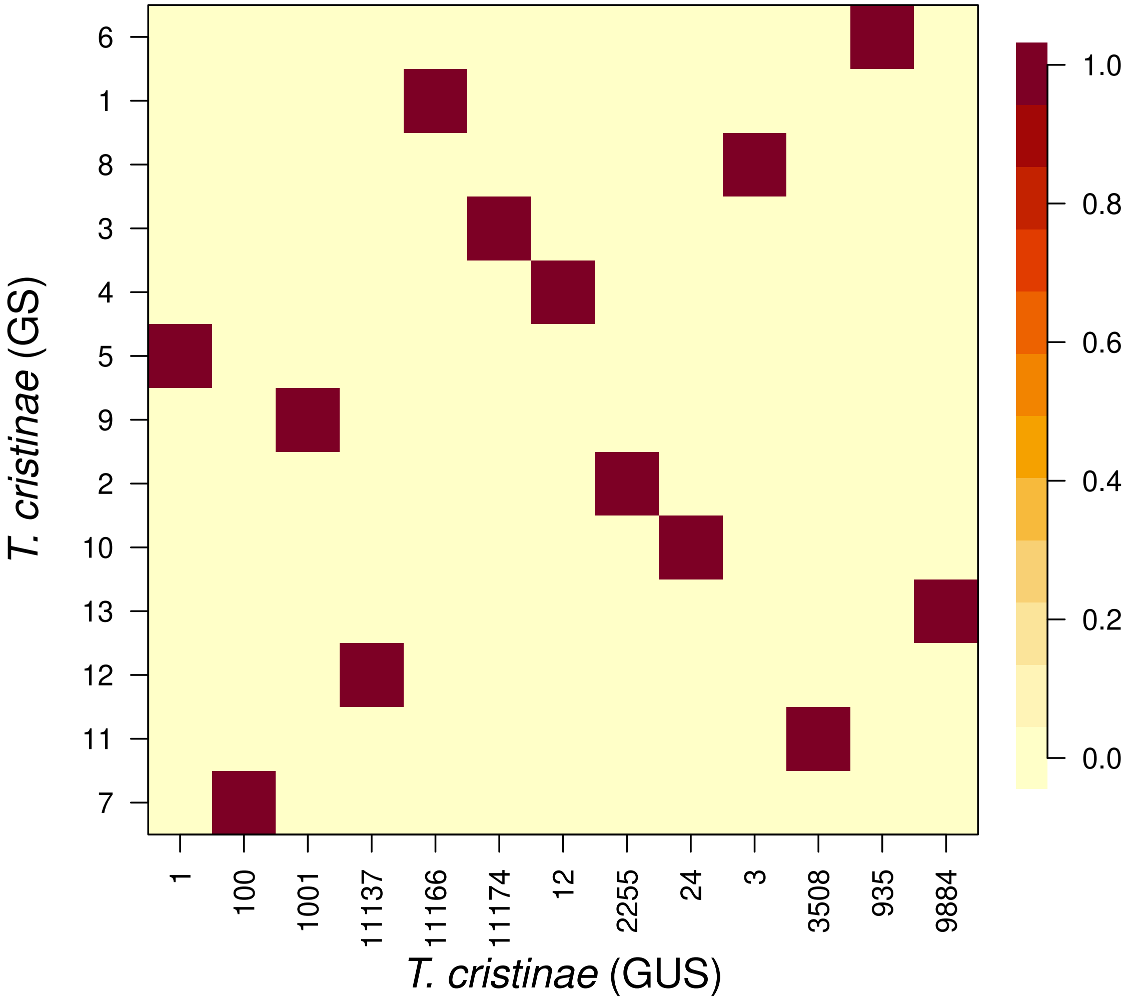

Figure S6: Correspondence between *T. cristinae* striped morph (GS) chromosome and scaffolds (chromosomes) for the green unstriped (GUS) morph. The heatmap shows the proportion of synteny blocks involving each striped morph chromosome that aligned to each of the green morph scaffolds.

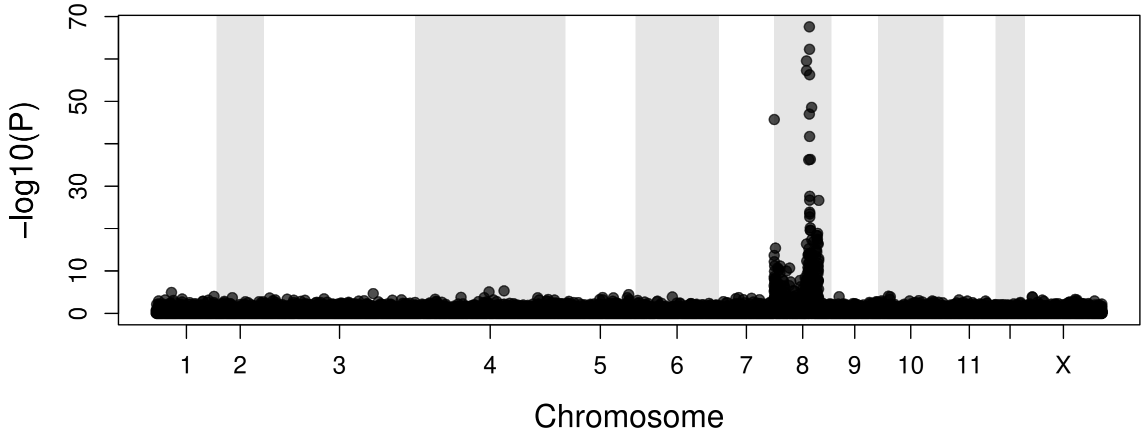

Figure S7: Genome-wide association mapping results for the green genome. This plot shows a Manhattan plot of the negative log10 P-value for the association between genome-wide SNPs and color pattern (green or striped). Points denote individual SNPs and shaded regions delimit chromosomes.

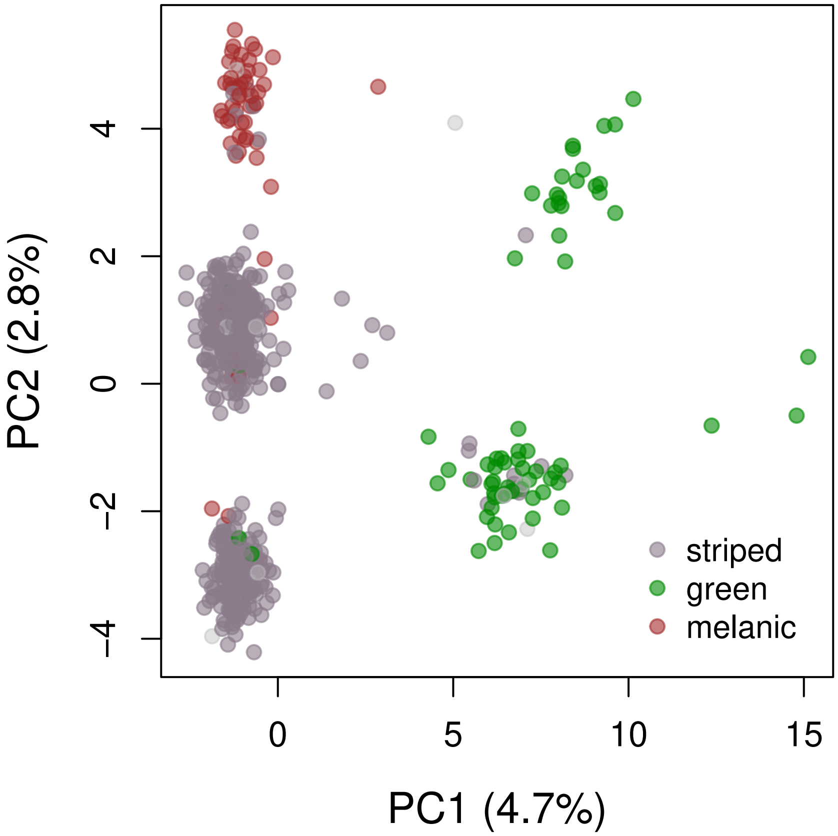

Figure S8: Statistical summary of genetic variation and clustering in the color-pattern loci. The scatterplot shows clustering of *T. cristinae* based on a principal components analysis (PCA) of the SNPs in the color-pattern loci. This is based on the 602 individuals from the FHA population. Here, the plot includes green, striped and melanic stick insects (compare to Figure 8B). Points denote PC1 and PC2 scores and are colored based on phenotype. Six clusters, which are related to color and color-pattern, are evident.

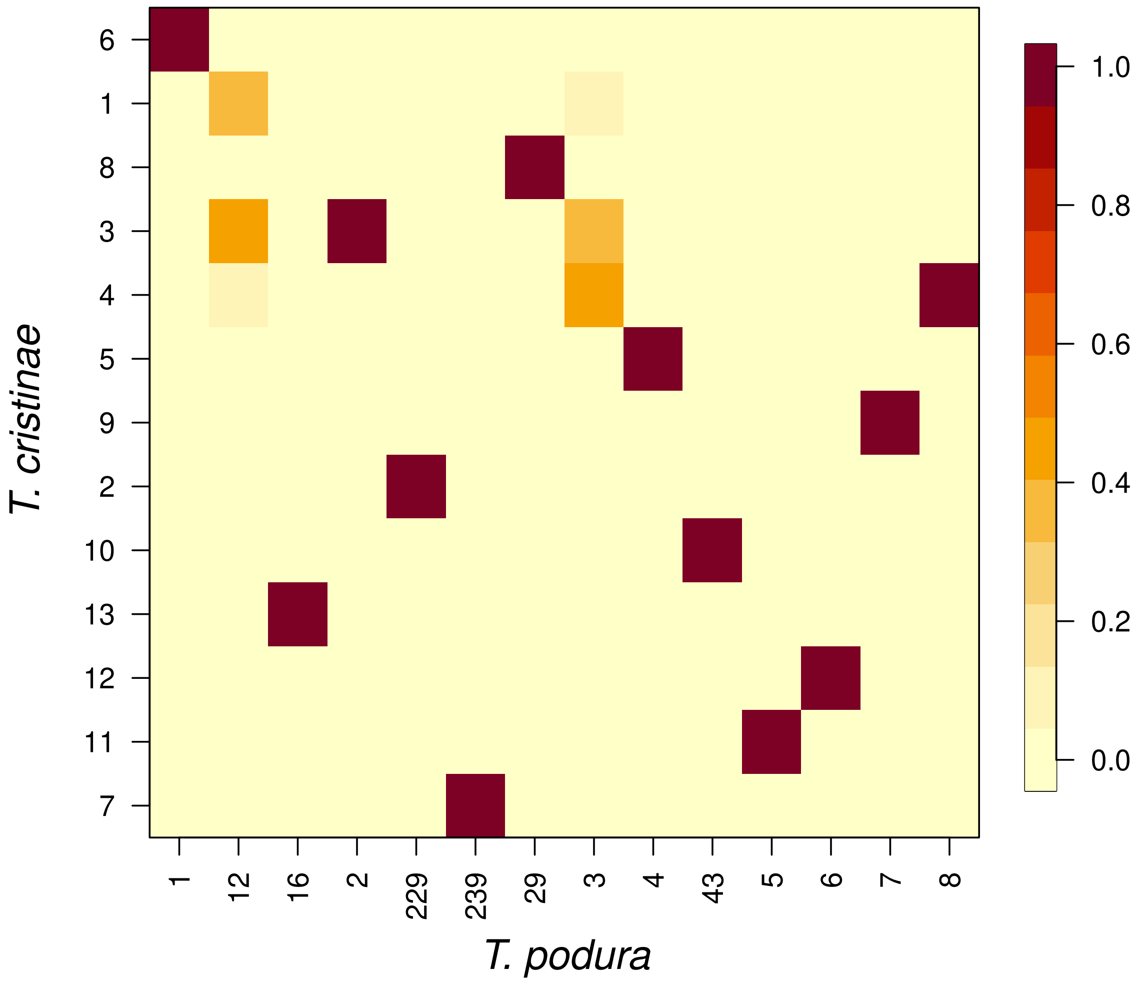

Figure S9: Heatmap shows the proportion synteny blocks on of each *T. podura* scaffold that aligned to each of the *T. cristinae* chromosomes. Results are shown for the striped morph.

Data S1: Database file containing *T. cristinae* morph count data. The database file (DatabaseS1.csv) contains 692 records. Fields (columns) provide information on the species, location, year, latitude (decimal degrees), longitude (decimal degrees), elevation, host (A = *Adenostoma*, C = *Ceanothus*), and counts of green unstriped (unstriped), green striped (striped), melanistic, and uncertain/intermediate (intermediate) morphs stick insects, along with the total count (total). Raw proportions from these counts are also reported. Lastly, climate data for each location, which are not analyzed in the current manuscript, are provided for a subsite of sites/years.

REFERENCES AND NOTES

1. Z. D. Blount, R. E. Lenski, J. B. Losos, Contingency and determinism in evolution: Replaying life's tape. *Science* **362**, eaam5979 (2018).

2. P. F. Colosimo, K. E. Hosemann, S. Balabhadra, G. Villarreal Jr, M. Dickson, J. Grimwood, J. Schmutz, R. M. Myers, D. Schluter, D. M. Kingsley, Widespread parallel evolution in sticklebacks by repeated fixation of ectodysplasin alleles. *Science* **307**, 1928–1933 (2005).

3. D. L. Stern, The genetic causes of convergent evolution. *Nat. Rev. Genet.* **14**, 751–764 (2013).

4. D. M. Weinreich, N. F. Delaney, M. A. DePristo, D. L. Hartl, Darwinian evolution can follow only very few mutational paths to fitter proteins. *Science* **312**, 111–114 (2006).

5. S. J. Gould, *Wonderful Life—The Burgess Shale and the Nature of History* (W. W. Norton and Company, 1990).

6. S. C. Morris, Evolution: Like any other science it is predictable. *Philos. Trans. R. Soc. B: Biol. Sci.* **365**, 133–145 (2010).

7. J. B. Losos, *Lizards in an Evolutionary Tree: Ecology and Adaptive Radiation of Anoles* (University of California Press, 2011), vol. **10**.

8. J. Arendt, D. Reznick, Convergence and parallelism reconsidered: What have we learned about the genetics of adaptation? *Trends Ecol. Evol.* **23**, 26–32 (2008).

9. J. B. Losos, *Improbable Destinies: How Predictable Is Evolution?* (Penguin, 2017).

10. T. van der Valk, P. Pečnerová, D. Díez-del-Molino, A. Bergström, J. Oppenheimer, S. Hartmann, G. Xenikoudakis, J. A. Thomas, M. Dehasque, E. Sağlıcan, F. R. Fidan, I. Barnes, S. Liu, M. Somel, P. D. Heintzman, P. Nikolskiy, B. Shapiro, P. Skoglund, M. Hofreiter, A. M. Lister, A. Götherström, L. Dalén, Million-year-old DNA sheds light on the genomic history of mammoths. *Nature* **591**, 265–269 (2021).

11. G. J. Vermeij, Historical contingency and the purported uniqueness of evolutionary innovations. *Proc. Natl. Acad. Sci. U.S.A.* **103**, 1804–1809 (2006).

12. J. R. Meyer, D. T. Dobias, J. S. Weitz, J. E. Barrick, R. T. Quick, R. E. Lenski, Repeatability and contingency in the evolution of a key innovation in phage lambda. *Science* **335**, 428–432 (2012).

13. J. Plucain, T. Hindré, M. Le Gac, O. Tenaillon, S. Cruveiller, C. Médigue, N. Leiby, W. R. Harcombe, C. J. Marx, R. E. Lenski, D. Schneider, Epistasis and allele specificity in the emergence of a stable polymorphism in *Escherichia coli*. *Science* **343**, 1366–1369 (2014).

14. M. Travisano, J. A. Mongold, A. F. Bennett, R. E. Lenski, Experimental tests of the roles of adaptation, chance, and history in Evolution. *Science* **267**, 87–90 (1995).

15. Z. Gompert, F. J. Messina, Genomic evidence that resource-based trade-offs limit host-range expansion in a seed beetle. *Evolution* **70**, 1249–1264 (2016).

16. W. R. Rice, E. E. Hostert, Laboratory Experiments on speciation: What have we learned in 40 years? *Evolution* **47**, 1637–1653 (1993).

17. M. Bosse, L. G. Spurgin, V. N. Laine, E. F. Cole, J. A. Firth, P. Gienapp, A. G. Gosler, K. McMahon, J. Poissant, I. Verhagen, M. A. M. Groenen, K. Van Oers, B. C. Sheldon, M. E. Visser, J. Slate, Recent natural selection causes adaptive evolution of an avian polygenic trait. *Science* **358**, 365–368 (2017).

18. A. Charmantier, R. H. McCleery, L. R. Cole, C. Perrins, L. E. B. Kruuk, B. C. Sheldon, Adaptive phenotypic plasticity in response to climate change in a wild bird population. *Science* **320**, 800–803 (2008).

19. P. R. Grant, B. R. Grant, Evolution of character displacement in Darwin's finches. *Science* **313**, 224–226 (2006).

20. P. R. Grant, B. R. Grant, Unpredictable evolution in a 30-year study of Darwin's finches. *Science* **296**, 707–711 (2002).

21. I. Hanski, Metapopulation dynamics. *Nature* **396**, 41–49 (1998).

22. H. B. D. Kettlewell, Selection experiments on industrial melanism in the Lepidoptera. *Heredity* **9**, 323–342 (1955).

23. M. C. Singer, C. Parmesan, Lethal trap created by adaptive evolutionary response to an exotic resource. *Nature* **557**, 238–241 (2018).

24. D. A. Marques, F. C. Jones, D. Palma, D. M. Kingsley, T. E. Reimchen, Experimental evidence for rapid genomic adaptation to a new niche in an adaptive radiation. *Nat. Ecol. Evol.* **2**, 1128–1138 (2018).

25. T. E. Reimchen, Predator-induced cyclical changes in lateral plate frequencies of *Gasterosteus*. *Behaviour* **132**, 1079–1094 (1995).

26. T. E. Reimchen, P. Nosil, Temporal variation in divergent selection on spine number in threespine stickleback. *Evolution* **56**, 2472–2483 (2002).

27. D. N. Reznick, H. Bryga, Life-history evolution in guppies (*Poecilia reticulata*): 1. Phenotypic and genetic changes in an introduction experiment. *Evolution* **41**, 1370–1385 (1987).

28. S. E. Johnston, J. Gratten, C. Berenos, J. G. Pilkington, T. H. Clutton-Brock, J. M. Pemberton, J. Slate, Life history trade-offs at a single locus maintain sexually selected genetic variation. *Nature* **502**, 93–95 (2013).

29. A. O. Bergland, E. L. Behrman, K. R. O'Brien, P. S. Schmidt, D. A. Petrov, Genomic evidence of rapid and stable adaptive oscillations over seasonal time scales in *Drosophila*. *PLOS Genet.* **10**, e1004775 (2014).

30. L. E. B. Kruuk, J. Slate, J. M. Pemberton, S. Brotherstone, F. Guinness, T. Clutton-Brock, Antler size in red deer: Heritability and selection but no evolution. *Evolution* **56**, 1683–1695 (2002).

31. R. D. H. Barrett, D. Schluter, Adaptation from standing genetic variation. *Trends Ecol. Evol.* **23**, 38–44 (2008).

32. G. L. Conte, M. E. Arnegard, C. L. Peichel, D. Schluter, The probability of genetic parallelism and convergence in natural populations. *Proc. R. Soc. B: Biol. Sci.* **279**, 5039–5047 (2012).

33. M. Bohutínská, C. L. Peichel, Divergence time shapes gene reuse during repeated adaptation. *Trends Ecol. Evol.* **39**, 396–407 (2024).

34. Z. D. Blount, C. Z. Borland, R. E. Lenski, Historical contingency and the evolution of a key innovation in an experimental population of *Escherichia coli*. *Proc. Natl. Acad. Sci.* **105**, 7899–7906 (2008).

35. S. Kryazhimskiy, D. P. Rice, E. R. Jerison, M. M. Desai, Global epistasis makes adaptation predictable despite sequence-level stochasticity. *Science* **344**, 1519–1522 (2014).

36. R. M. Varney, D. I. Speiser, J. T. Cannon, M. A. Aguilar, D. J. Eernisse, T. H. Oakley, A morphological basis for path-dependent evolution of visual systems. *Science* **383**, 983–987 (2024).

37. S. Yeaman, Local adaptation by alleles of small effect. *Am. Natural.* **186**, S74–S89 (2015).

38. J. F. Storz, Causes of molecular convergence and parallelism in protein evolution. *Nat. Rev. Genet.* **17**, 239–250 (2016).

39. B. J. Crespi, C. P. Sandoval, Phylogenetic evidence for the evolution of ecological specialization in Timema walking-sticks. *J. Evol. Biol.* **13**, 249–262 (2000).

40. A. A. Comeault, S. M. Flaxman, R. Riesch, E. Curran, V. Soria-Carrasco, Z. Gompert, T. E. Farkas, M. Muschick, T. L. Parchman, T. Schwander, J. Slate, P. Nosil, Selection on a genetic polymorphism counteracts ecological speciation in a stick insect. *Curr. Biol.* **25**, 1975–1981 (2015).

41. P. Nosil, Divergent host plant adaptation and reproductive isolation between ecotypes of *Timema cristinae* walking sticks. *Am. Nat.* **169**, 151–162 (2007).

42. P. Nosil, B. J. Crespi, Experimental evidence that predation promotes divergence in adaptive radiation. *Proc. Natl. Acad. Sci. U.S.A.* **103**, 9090–9095 (2006).

43. C. P. Sandoval, Differential visual predation on morphs of *Timema cristinae* (Phasmatodeae:Timemidae) and its consequences for host range. *Biol. J. Linn. Soc.* **52**, 341–356 (1994).

44. P. Nosil, B. J. Crespi, C. P. Sandoval, Host-plant adaptation drives the parallel evolution of reproductive isolation. *Nature* **417**, 440–443 (2002).

45. P. Nosil, R. Villoutreix, C. F. de Carvalho, T. E. Farkas, V. Soria-Carrasco, J. L. Feder, B. J. Crespi, Z. Gompert, Natural selection and the predictability of evolution in Timema stick insects. *Science* **359**, 765–770 (2018).

46. C. P. Sandoval, The effects of the relative geographic scales of gene flow and selection on morph frequencies in the walking-stick *Timema cristinae*. *Evolution* **48**, 1866–1879 (1994).

47. D. Lindtke, K. Lucek, V. Soria-Carrasco, R. Villoutreix, T. E. Farkas, R. Riesch, S. R. Dennis, Z. Gompert, P. Nosil, Long-term balancing selection on chromosomal variants associated with crypsis in a stick insect. *Mol. Ecol.* **26**, 6189–6205 (2017).

48. A. B. Bond, The evolution of color polymorphism: Crypticity, searching images, and apostatic selection. *Annu. Rev. Ecol. Evol. Syst.* **38**, 489–514 (2007).

49. A. B. Bond, A. C. Kamil, Visual predators select for crypticity and polymorphism in virtual prey. *Nature* **415**, 609–613 (2002).

50. J. A. Allen, B. Clarke, Evidence for apostatic selection by wild passerines. *Nature* **220**, 501–502 (1968).

51. J. A. Allen, Further evidence for apostatic selection by wild passerine birds—9:1 experiments. *Heredity* **36**, 173–180 (1976).

52. C. Sandoval, Persistence of a walking-stick population (Phasmatoptera: Timematodea) after a wildfire. *Southw. Natural.* **45**, 123–127 (2000).

53. P. Nosil, B. J. Crespi, C. P. Sandoval, Reproductive isolation driven by the combined effects of ecological adaptation and reinforcement. *Proc. R. Soc. Lond. B: Biol. Sci.* **270**, 1911–1918 (2003).

54. P. Nosil, *Ecological Speciation* (Oxford Univ. Press, 2012).

55. R. Villoutreix, C. F. de Carvalho, V. Soria-Carrasco, D. Lindtke, M. De-la-Mora, M. Muschick, J. L. Feder, T. L. Parchman, Z. Gompert, P. Nosil, Large-scale mutation in the evolution of a gene complex for cryptic coloration. *Science* **369**, 460–466 (2020).

56. R. Riesch, M. Muschick, D. Lindtke, R. Villoutreix, A. A. Comeault, T. E. Farkas, K. Lucek, E. Hellen, V. Soria-Carrasco, S. R. Dennis, C. F. De Carvalho, R. J. Safran, C. P. Sandoval, J. Feder, R. Gries, B. J. Crespi, G. Gries, Z. Gompert, P. Nosil, Transitions between phases of genomic differentiation during stick-insect speciation. *Nat. Ecol. Evol.* **1**, 82 (2017).

57. R. C. Lewontin, A general method for investigating the equilibrium of gene frequency in a population. *Genetics* **43**, 419–434 (1958).

58. L.-M. Chevin, Z. Gompert, P. Nosil, Frequency dependence and the predictability of evolution in a changing environment. *Evol. Lett.* **6**, 21–33 (2022).

59. J. K. Goldberg, C. M. Lively, S. R. Sternlieb, G. Pintel, J. D. Hare, M. B. Morrissey, L. F. Delph, Herbivore-mediated negative frequency-dependent selection underlies a trichome dimorphism in nature. *Evol. Lett.* **4**, 83–90 (2020).

60. D. I. Bolnick, P. Nosil, Natural selection in populations subject to a migration load. *Evolution* **61**, 2229–2243 (2007).

61. E. I. Svensson, J. Abbott, R. Härdling, Female polymorphism, frequency dependence, and rapid evolutionary dynamics in natural populations. *Am. Nat.* **165**, 567–576 (2005).

62. E. I. Svensson, T. Connallon, How frequency-dependent selection affects population fitness, maladaptation and evolutionary rescue. *Evol. Appl.* **12**, 1243–1258 (2019).

63. P. Jay, T. G. Aubier, M. Joron, The interplay of local adaptation and gene flow may lead to the formation of supergenes. *Mol. Ecol.* e17297 (2024).

64. P. Nosil, Reproductive isolation caused by visual predation on migrants between divergent environments. *Proc. R. Soc. Lond. B: Biol. Sci.* **271**, 1521–1528 (2004).

65. Z. Gompert, A. A. Comeault, T. E. Farkas, J. L. Feder, T. L. Parchman, C. A. Buerkle, P. Nosil, Experimental evidence for ecological selection on genome variation in the wild. *Ecol. Lett.* **17**, 369–379 (2014).

66. Z. Gompert, J. L. Feder, P. Nosil, The short-term, genome-wide effects of indirect selection deserve study: A response to Charlesworth and Jensen (2022). *Mol. Ecol.* **31**, 4444–4450 (2022).

67. V. Soria-Carrasco, Z. Gompert, A. Comeault, T. E. Farkas, T. L. Parchman, J. S. Johnston, C. A. Buerkle, J. L. Feder, J. Bast, T. Schwander, S. P. Egan, B. J. Crespi, P. Nosil, Stick insect genomes reveal natural selection's role in parallel speciation. *Science* **344**, 738–42 (2014).

68. S. Chaturvedi, Z. Gompert, J. L. Feder, O. G. Osborne, M. Muschick, R. Riesch, V. Soria-Carrasco, P. Nosil, Climatic similarity and genomic background shape the extent of parallel adaptation in *Timema* stick insects. *Nat. Ecol. Evol.* **6**, 1952–1964 (2022).

69. L. S. Zamorano, Z. Gompert, E. A. Fronhofer, J. L. Feder, P. Nosil, A stabilizing eco-evolutionary feedback loop in the wild. *Curr. Biol.* **33**, 3272–3278.e3 (2023).

70. A. A. Comeault, C. Ferreira, S. Dennis, V. Soria-Carrasco, P. Nosil, Color phenotypes are under similar genetic control in two distantly related species of *Timema* stick insect. *Evolution* **70**, 1283–1296 (2016).

71. C. P. Sandoval, P. Nosil, Counteracting selective regimes and host preference evolution in ecotypes of two species of walking-sticks. *Evolution* **59**, 2405–2413 (2005).

72. P. Nosil, V. Soria-Carrasco, R. Villoutreix, M. De-la-Mora, C. F. de Carvalho, T. Parchman, J. L. Feder, Z. Gompert, Complex evolutionary processes maintain an ancient chromosomal inversion. *Proc. Natl. Acad. Sci. U.S.A.* **120**, e2300673120 (2023).

73. S. Wright, The shifting balance theory and macroevolution. *Annu. Rev. Genet.* **16**, 1–20 (1982).

74. Y. F. Chan, M. E. Marks, F. C. Jones, G. Villarreal, M. D. Shapiro, S. D. Brady, A. M. Southwick, D. M. Absher, J. Grimwood, J. Schmutz, R. M. Myers, D. Petrov, B. Jónsson, D. Schluter, M. A. Bell, D. M. Kingsley, Adaptive evolution of pelvic reduction in sticklebacks by recurrent deletion of a Pitx1 enhancer. *Science* **327**, 302–305 (2010).

75. K. T. Xie, G. Wang, A. C. Thompson, J. I. Wucherpfennig, T. E. Reimchen, A. D. C. MacColl, D. Schluter, M. A. Bell, K. M. Vasquez, D. M. Kingsley, DNA fragility in the parallel evolution of pelvic reduction in stickleback fish. *Science* **363**, 81–84 (2019).

76. K.-W. Kim, R. De-Kayne, I. J. Gordon, K. S. Omufwoko, D. J. Martins, R. Ffrench-Constant, S. H. Martin, Stepwise evolution of a butterfly supergene via duplication and inversion. *Philosop. Trans. R. Soc. B: Biol. Sci.* **377**, 20210207 (2022).

77. D. J. Funk, Isolating a role for natural selection in speciation: Host adaptation and sexual isolation in *Neochlamisus bebbianae* leaf beetles. *Evolution* **52**, 1744–1759 (1998).

78. D. Schluter, Evidence for ecological speciation and its alternative. *Science* **323**, 737–741 (2009).

79. D. Schluter, *The Ecology of Adaptive Radiation* (Oxford Univ. Press, 2000).

80. P. Nosil, T. H. Vines, D. J. Funk, Reproductive isolation caused by natural selection against immigrants from divergent habitats. *Evolution* **59**, 705–719 (2005).

81. P. Nosil, B. J. Crespi, C. P. Sandoval, M. Kirkpatrick, Migration and the genetic covariance between habitat preference and performance. *Am. Nat.* **167**, E66–E78 (2006).

82. P. Nosil, C. P. Sandoval, B. J. Crespi, The evolution of host preference in allopatric vs. parapatric populations of *Timema cristinae* walking-sticks. *J. Evol. Biol.* **19**, 929–942 (2006).

83. P. Nosil, J. L. Feder, S. M. Flaxman, Z. Gompert, Tipping points in the dynamics of speciation. *Nat. Ecol. Evol.* **1**, 1 (2017).

84. M. Scheffer, S. R. Carpenter, T. M. Lenton, J. Bascompte, W. Brock, V. Dakos, J. Van de Koppel, I. A. Van de Leemput, S. A. Levin, E. H. Van Nes, M. Pascual, Anticipating critical transitions. *Science* **338**, 344–348 (2012).

85. R. C. Lewontin, *The Genetic Basis of Evolutionary Change* (Columbia Univ. Press, 1974).

86. P. Nosil, Z. Gompert, T. E. Farkas, A. A. Comeault, J. L. Feder, C. S. Buerkle, T. L. Parchman, Genomic consequences of multiple speciation processes in a stick insect. *Proc. R. Soc. B: Biol. Sci.* **279**, 5058–5065 (2012).

87. J. Armstrong, G. Hickey, M. Diekhans, I. T. Fiddes, A. M. Novak, A. Deran, Q. Fang, D. Xie, S. Feng, J. Stiller, D. Genereux, J. Johnson, V. D. Marinescu, J. Alföldi, R. S. Harris, K. Lindblad-Toh, D.

Haussler, E. Karlsson, E. D. Jarvis, G. Zhang, B. Paten, Progressive Cactus is a multiple-genome aligner for the thousand-genome era. *Nature* **587**, 246–251 (2020).

88. B. Paten, D. Earl, N. Nguyen, M. Diekhans, D. Zerbino, D. Haussler, Cactus: Algorithms for genome multiple sequence alignment. *Genome Res.* **21**, 1512–1528 (2011).

89. A. F. Smit, Repeat-Masker Open-3.0 (2004); www.repeatmasker.org.

90. K. Krasheninnikova, M. Diekhans, J. Armstrong, A. Dievskii, B. Paten, S. O'Brien, HalSynteny: A fast, easy-to-use conserved synteny block construction method for multiple whole-genome alignments. *GigaScience* **9**, giaa047 (2020).

91. H. Li, R. Durbin, Fast and accurate short read alignment with Burrows-Wheeler transform. *Bioinformatics* **25**, 1754–1760 (2009).

92. H. Li, B. Handsaker, A. Wysoker, T. Fennell, J. Ruan, N. Homer, G. Marth, G. Abecasis, R. Durbin, The sequence alignment/map format and SAMtools. *Bioinformatics* **25**, 2078–2079 (2009).

93. X. Zhou, M. Stephens, Genome-wide efficient mixed-model analysis for association studies. *Nat. Genet.* **44**, 821–824 (2012).

94. H. Li, P. Ralph, Local PCA shows how the effect of population structure differs along the genome. *Genetics* **211**, 289–304 (2019).

95. M. Todesco, G. L. Owens, N. Bercovich, J. S. Légaré, S. Soudi, D. O. Burge, K. Huang, K. L. Ostevik, E. B. M. Drummond, I. Imerovski, K. Lande, M. A. Pascual-Robles, M. Nanavati, M. Jahani, W. Cheung, S. E. Staton, S. Muños, R. Nielsen, L. A. Donovan, J. M. Burke, S. Yeaman, L. H. Rieseberg, Massive haplotypes underlie ecotypic differentiation in sunflowers. *Nature* **584**, 602–607 (2020).

96. W. J. Ewens, *Mathematical Population Genetics I. Theoretical Introduction*, Interdisciplinary Applied Mathematics (Springer, 2004).

97. S. Wright, *Evolution and the Genetics of Populations: A Treatise in Four, Volume 4: Variability Within and Among Natural Populations* (University of Chicago Press, 1978).

98. M. Plummer, rjags: Bayesian graphical models using MCMC, R package version 4-14 (2023); https://CRAN.R-project.org/package=rjags.

99. M. Plummer, N. Best, K. Cowles, K. Vines, CODA: Convergence diagnosis and output analysis for MCMC. *R News* **6**, 7–11 (2006).

100. R. M. Neal, "MCMC using Hamiltonian dynamics" in *Handbook of Markov Chain Monte Carlo* (CRC Press 2011), p. 113–160.

101. Stan Development Team, RStan: The R interface to Stan, R package version 2.21.8 (2023); https://mc-stan.org/.

102. M. D. Hoffman, A. Gelman, The No-U-Turn sampler: Adaptively setting path lengths in Hamiltonian Monte Carlo. *J. Mach. Learn. Res.* **15**, 1593–1623 (2014).

103. Z. Gompert, S. M. Flaxman, J. L. Feder, L.-M. Chevin, P. Nosil, Laplace's demon in biology: Models of evolutionary prediction. *Evolution* **76**, 2794–2810 (2022).

104. S. Rice, *Evolutionary Theory: Mathematical and Conceptual Foundations* (Sinauer Associates, 2004).

105. A. Vehtari, A. Gelman, J. Gabry, Practical Bayesian model evaluation using leave-one-out cross-validation and WAIC. *Statist. Comput.* **27**, 1413–1432 (2017).

106. N. H. Putnam, B. L. O'Connell, J. C. Stites, B. J. Rice, M. Blanchette, R. Calef, C. J. Troll, A. Fields, P. D. Hartley, C. W. Sugnet, D. Haussler, D. S. Rokhsar, R. E. Green, Chromosome-scale shotgun assembly using an in vitro method for long-range linkage. *Genome Res.* **26**, 342–350 (2016).

107. E. Lieberman-Aiden, N. L. Van Berkum, L. Williams, M. Imakaev, T. Ragoczy, A. Telling, I. Amit, B. R. Lajoie, P. J. Sabo, M. O. Dorschner, R. Sandstrom, B. Bernstein, M. A. Bender, M. Groudine, A. Gnirke, J. Stamatoyannopoulos, L. A. Mirny, E. S. Lander, J. Dekker, Comprehensive mapping of long-range interactions reveals folding principles of the human genome. *Science* **326**, 289–293 (2009).

108. D. Harte, HiddenMarkov: Hidden Markov Models, Statistics Research Associates, Wellington, R package version 1.8-13 (2021); www.statsresearch.co.nz/dsh/sslib/.

109. L. E. Baum, T. Petrie, G. Soules, N. Weiss, A maximization technique occurring in the statistical analysis of probabilistic functions of Markov chains. *Ann. Math. Stat.* **41**, 164–171 (1970).

110. G. D. Forney, The Viterbi algorithm. *Proc. IEEE* **61**, 268–278 (1973).

111. J. Ruan, H. Li, Fast and accurate long-read assembly with wtdbg2. *Nat. Methods* **17**, 155–158 (2020).

112. D. R. Laetsch, M. L. Blaxter, BlobTools: Interrogation of genome assemblies. *F1000Res.* **6**, 1287 (2017).

113. D. Guan, S. A. McCarthy, J. Wood, K. Howe, Y. Wang, R. Rubin Identifying and removing haplotypic duplication in primary genome assemblies. *Bioinformatics* **36**, 2896–2898 (2020).
